# Comparative population genomics reveals genetic divergence and selection in lotus, *Nelumbo nucifera*

**DOI:** 10.1186/s12864-019-6376-8

**Published:** 2020-02-11

**Authors:** Ye Li, Feng-Lin Zhu, Xing-Wen Zheng, Man-Li Hu, Chen Dong, Ying Diao, You-Wei Wang, Ke-Qiang Xie, Zhong-Li Hu

**Affiliations:** 1 State Key Laboratory of Hybrid Rice, Lotus Engineering Research Center of Hubei Province, College of Life Sciences, Wuhan University, Wuhan, 430072 People’s Republic of China; 2 Institute of Traditional Chinese Medicine and Natural Products, School of Pharmaceutical Sciences, Wuhan University, Wuhan, 430071 People’s Republic of China; 3 Guangchang Research School of White Lotus, Guangchang, 344900 People’s Republic of China; 4 College of Landscape Architecture and Life Science / Institute of Special Plants, Chongqing University of Arts and Sciences, Chongqing, 402160 People’s Republic of China

**Keywords:** *Nelumbo nucifera*, Whole-genome resequencing, Genome variation, Domestication

## Abstract

**Background:**

Lotus (*Nelumbo nucifera*) is an aquatic plant with important agronomic, horticulture, art and religion values. It was the basal eudicot species occupying a critical phylogenetic position in flowering plants. After the domestication for thousands of years, lotus has differentiated into three cultivated types -flower lotus, seed lotus and rhizome lotus. Although the phenotypic and genetic differentiations based on molecular markers have been reported, the variation on whole-genome level among the different lotus types is still ambiguous.

**Results:**

In order to reveal the evolution and domestication characteristics of lotus, a total of 69 lotus accessions were selected, including 45 cultivated accessions, 22 wild sacred lotus accessions, and 2 wild American lotus accessions. With Illumina technology, the genomes of these lotus accessions were resequenced to > 13× raw data coverage. On the basis of these genomic data, 25 million single-nucleotide polymorphisms (SNPs) were identified in lotus. Population analysis showed that the rhizome and seed lotus were monophyletic and genetically homogeneous, whereas the flower lotus was biphyletic and genetically heterogeneous. Using population SNP data, we identified 1214 selected regions in seed lotus, 95 in rhizome lotus, and 37 in flower lotus. Some of the genes in these regions contributed to the essential domestication traits of lotus. The selected genes of seed lotus mainly affected lotus seed weight, size and nutritional quality. While the selected genes were responsible for insect resistance, antibacterial immunity and freezing and heat stress resistance in flower lotus, and improved the size of rhizome in rhizome lotus, respectively.

**Conclusions:**

The genome differentiation and a set of domestication genes were identified from three types of cultivated lotus- flower lotus, seed lotus and rhizome lotus, respectively. Among cultivated lotus, flower lotus showed the greatest variation. The domestication genes may show agronomic importance via enhancing insect resistance, improving seed weight and size, or regulating lotus rhizome size. The domestication history of lotus enhances our knowledge of perennial aquatic crop evolution, and the obtained dataset provides a basis for future genomics-enabled breeding.

## Background

*Nelumbo* Adans., the earliest originating genus among angiosperms, is a surviving living fossil that experienced quaternary glaciation, with an evolutionary history of approximately 135 million years [1, 2]. At present, there are two species in *Nelumbo*. *N. nucifera* Gaertn. (Sacred lotus) possesses white or red flowers and is distributed in Asia and Northern Oceania, whereas *N. lutea* (Willd.) Pers. (American lotus) produces yellow flowers and is distributed across North America [3].

In the long course of human civilization, people have noted the unique biological characteristics of lotus and conferred upon the plant corresponding cultural connotation. As such, lotus has become a culturally important plant and has been praised for thousands of years in numerous artworks, including poetry, music, dance, and painting. The best-known feature of lotus is its water-repellent self-cleaning function, which maintains its beauty and cleanliness, despite growing in dirty ponds [4–7]. Thus, lotus is considered a holy flower in Buddhism, Hinduism, and Taoism and symbolizes grace, purity, and serenity. Another characteristic of lotus is multi-seed production. The seeds exhibit strong vitality, allowing them to germinate and grow thousands of years after they were produced [8–11]. Such robust and continual vitality is highly respected, and lotus seeds are regarded as a traditional wedding keepsake in China and a symbol of generational continuity [12, 13]. .

There has been great progress in animal and plant domestication in the past 13 thousand years of human history, which has contributed to the majority of current human food sources and has been required for the ascent of civilization. Moreover, domestication has modified the distribution of the world’s population. Through genomic variation analysis of food crops such as rice [14], corn [15], sorghum [16], soybean [17], tomato [18], cucumber [19], and peach [20], scholars have observed the effects of human domestication on plant evolution. Since used as a food for over 7000 years in Asia, the cultivated varieties of *N. nucifera* have differentiated into three types: rhizome lotus, seed lotus, and flower lotus [21]. The variety dominated by the edible underground stem is known as rhizome lotus. This variety produces few flowers and a considerably enlarged underground stem with stored starch. The variety dominated by the edible seeds is known as seed lotus. This type of lotus has dense flowers and generates larger and more numerous seeds than are produced by other varieties. The ornamental variety is known as flower lotus and exhibits beautiful flowers with rich patterns, colors and size variation. Although lotus plants exhibit two modes of reproduction (i.e., sexual reproduction and asexual reproduction), lotus varieties are generally propagated via vegetative reproduction through rhizomes.

Genome sequences of lotus have been published, laying a foundation for the analysis of genome variation [21, 22]. Up to date, few accessions were used to analysis the whole-genome variation of lotus [23]. Knowledge of genetic divergence and selection of their genomes during its domestication remains limited. In this study, we performed whole-genome resequencing and comparative analysis of 69 lotus accessions. Our results reveal the evolution and domestication characteristics of lotus, helping us to further understand this ancient and elegant plant. This paper also provides a reference for the further development and application of lotus varieties.

## Results

### Genome sequencing and mapping

**Fig. 1:**
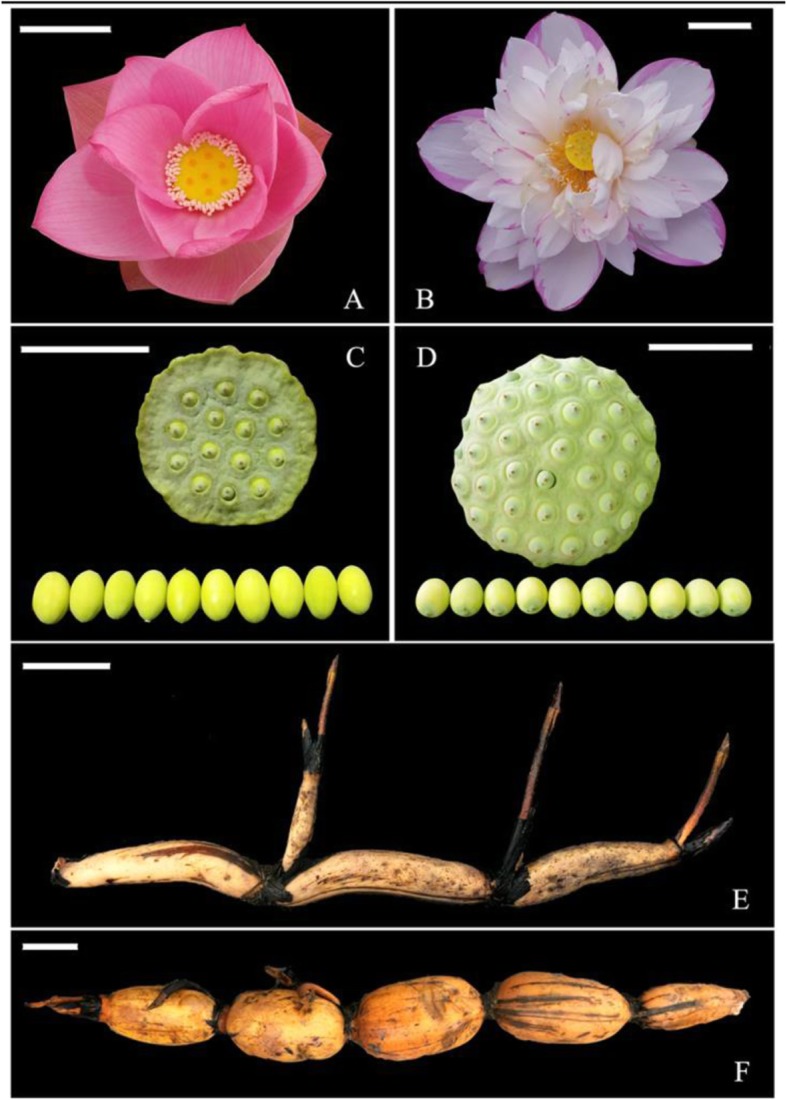
Morphology of the four lotus groups. Flower (**a**), seeds (**c**) and rhizome (**e**) of lotus accession W06 (wild sacred lotus). A flower of lotus accession F10 (flower lotus), seeds of lotus accession S19 (seed lotus), and a rhizome of lotus accession R11 (rhizome lotus) are shown in figures (**b**), **d** and **f** respectively. Bar indicates 10 cm

In this study, 69 accessions were selected, including 67 *N. nucifera* accessions (11 flower lotus, 13 rhizome lotus, 21 seed lotus, and 22 wild lotus) and two *N. lutea* (American lotus) accessions (Fig. 1, Additional file 1: Table S1, Additional file 6: Figure S1). The cultivated varieties selected in this study were among the most representative materials showing typical phenotypic differentiation or the most widely planted materials with high commercial value. Wild materials were collected from the natural lotus distribution regions of China, Thailand, Indonesia, and the USA.

Using Illumina HiSeqTM 2000 sequencing, we obtained 807 Gb of clean data. Compared with the reference genome of ‘China Antique’ lotus, the average mapping rate for the sequenced group samples was approximately 87.19%; the average genome sequencing depth was 13.54×; and the average coverage rate was approximately 98.34% (see Additional file 1: Table S1). The mapping rate in different accessions varied from 84.00 to 88.58%. The mapping rates for the two American lotus accessions were lowest, at 84.00 and 84.06%. The average mapping rate was 87.68% in wild sacred lotus. The average mapping rates for rhizome lotus, seed lotus, and flower lotus were 87.74, 86.77, and 87.61%, respectively. The observed differences in mapping rates were caused by the divergence between the sequenced genotypes and the reference genome of the sacred lotus variety ‘China Antique’.

The rates of heterozygosity in the different lotus groups varied greatly (see Additional file 1: Table S1), ranging from 0.14–0.73% (average, 0.45%) for flower lotus; 0.08–0.54% (average, 0.24%) for seed lotus; and 0.10–0.28% (average, 0.17%) for rhizome lotus. Most of the wild sacred lotus accessions showed low heterozygosity (Het rate (%) ≤ 0.1%), and the average heterozygosity was 0.14%. The heterozygosity values for the two American lotus accessions were 0.25 and 0.37%, respectively, which is similar to a previous report [21].

### Variation across the lotus genome

Using a strict pipeline, we identified 25,475,287 single-nucleotide polymorphisms (SNPs), with 27,422 SNPs per megabase on average; 2,753,718 indels (short insertions and deletions ranging from 1 to 5 bp in length), with 4732 indels per megabase; and 818,504 structural variations (SVs, > 5 bp) on average, with an average of 881 SVs per megabase (Table 1, see Additional file 2: Table S2 and Additional file 3: Table S3).

**Table 1:** Summary of single-nucleotide polymorphisms in lotus accessions

Groups	n.	Total	Private	θπ (10^− 3^)	Region												
					UTR3	UTR5	UTR5;UTR3	Intergenic	Intronic	CDS							
										Nonsynonymous	Stopgain	Stoploss	Synonymous	Unknown	Ratio of Nonsyn/Syn	Total	genes
Flower lotus	11	8,991,192	206,476	3.52	42,381	36,468	21	6,880,906	1,881,376	84,878	1939	287	60,138	2798	1.41	150,040	21,102
Seed lotus	21	8,161,881	469,267	2.46	34,590	29,459	23	6,321,473	1,650,468	71,889	1781	283	49,470	2445	1.45	125,868	21,197
Rhizome lotus	13	6,590,716	87,758	1.92	28,419	24,193	20	5,094,179	1,342,102	57,535	1313	237	40,641	2077	1.42	101,803	19,796
Wild sacred lotus	22	8,046,985	756,575	1.87	34,975	30,055	23	6,219,232	1,634,652	73,500	1783	277	50,018	2470	1.47	128,048	21,437
American lotus	2	18,504,122	13,957,952	–	118,767	103,748	170	13,611,092	4,289,740	211,456	4174	573	158,475	5927	1.33	380,605	23,839
Total	69	25,475,287	–	4.24	146,879	127,592	190	19,103,583	5,614,715	211,641	6042	745	196,050	7850	1.39	482,328	24,180

The accuracy of the SNPs and the genotyping inferences was estimated to be ~ 97.38–99.73% via Polymerase Chain Reaction (PCR) and Sanger sequencing (see Additional file 4: Table S4 and Additional file 5: Table S5). This result is consistent with previous resequencing results, where the SNP calling accuracy was found to be ~ 95–99% [14, 17, 24, 25]. Thus, our results met the requirements for further data mining and analysis.

A total of 74.99% (19,103,583) of the detected SNPs were distributed across intergenic regions, and 1.89% (482,328) of the detected SNPs were distributed across coding sequences (CDSs), covering 24,180 genes. Within untranslated regions (UTRs), 145,937 SNPs were distributed in 3′UTR, exceeding the 126,003 SNPs distributed in the 5′UTR (Table 1). In CDS regions, 211,641 were nonsynonymous and 196,050 synonymous SNPs, with a nonsynonymous/synonymous ratio of 1.39. This result was similar to those reported for soybean [1.37] [17], peach [1.31] [20], and rice [1.29] [14], but was slightly higher than in sorghum [1.0] [16] and *Arabidopsis* [0.83] [26]). Moreover, 6042 SNPs causing stop gains and 745 causing stop losses were found in CDS regions, possibly affecting gene expression.

Among the detected indels, 72.48% (1,995,912) were distributed across intergenic regions; 24.96% (687,414) were distributed across intronic regions; and 0.65% (18,046) were distributed in coding regions, affecting 7471 genes (see Additional file 2: Table S2). Among these sequences, the indels distributed in UTRs were more numerous than those in coding regions. A total of 7032 indels in CDSs causing frameshift deletions and 4560 causing frameshift insertions with possible alteration of gene expresion. Deletions, insertions, duplication, and inversions accounted for 63.3% (518,454), 31.8% (259,970), 3.3% (27,036), and 1.6% (13,044) of the detected SVs(> 5 bp), respectively (see Additional file 3: Table S3).

### Polymorphisms in the wild and three cultivated lotus groups

Flower lotus, seed lotus, rhizome lotus, wild sacred lotus, and American lotus differed greatly in terms of the identified SNP numbers. Some SNPs were shared among the five groups, whereas some were unique to one group. American lotus presented the highest number of SNPs (18,504,122; 73.64%), followed by flower lotus (8,991,192; 35.29%), seed lotus (8,161,881; 32.04%), wild sacred lotus (8,046,985; 31.59%), and rhizome lotus, which exhibited the lowest number of SNPs (6,590,716; 25.87%) (Table 1, Additional file 7: Figure S2). A total of 2,044,674 SNPs were shared by the five groups, and 220,145 SNPs were shared by the three cultivated lotus groups. Each group contained a substantial number of specific SNPs. American lotus group possessed the most unique SNPs (13,957,952), followed by wild sacred lotus (756,575), seed lotus (469,267), flower lotus (206,476), and rhizome lotus (87,758), which exhibited the fewest SNPs. Independent and shared SNPs reflected uniqueness and commonality, respectively, among the groups.

The SNP distribution in the genome varied between the different groups (Table 1). Among the four groups of *N. nucifera*, flower lotus displayed the largest number of SNPs distributed in intergenic, UTR, intronic, and CDS regions. However, rhizome lotus exhibited the fewest number of SNPs. The nonsynonymous/synonymous ratio for the *N. lutea* genome was lowest (1.33), while that for wild sacred lotus was highest (1.47), which was slightly higher than for the cultivated groups (rhizome lotus [1.42], flower lotus [1.41], and seed lotus [1.45]).

Tajima’s θπ was used to evaluate genetic polymorphism (Table 1). In *N. nucifera*, flower lotus showed the highest diversity (θπ (10^− 3^) = 3.52), followed by seed lotus (θπ (10^− 3^) = 2.46), and rhizome lotus ((θπ (10^− 3^) = 1.92). Moreover, wild sacred lotus (θπ (10^− 3^) = 1.87) presented slightly lower polymorphism level than that of rhizome lotus.

Indels greatly varied among the different groups (see Additional file 2: Table S2). The majority of indels (2,017,540; 73.27%) were found in American lotus, which also presented the most unique indels (1,672,002; 60.72%). In *N. nucifera*, the percentage of indels was reduced, ranging from 29.58% (flower lotus) to 20.80% (rhizome lotus), and the percentage of unique indels was reduced even more sharply, to 2.76% in wild lotus and 0.41% in rhizome lotus. Approximately 4.57% of indels were shared by the five groups, and 0.91% were shared by the cultivated population (see Additional file 8: Figure S3). The indels were mainly located in intergenic and intronic regions in all groups. The number of indels located in CDS regions was highest in *N. lutea*, followed by flower lotus and wild sacred lotus; seed lotus displayed an intermediate number, and rhizome lotus exhibited the fewest. The number of indels in intergenic, intronic, UTR3 and UTR5 regions showed a trend similar to that in CDS regions among the five groups.

SVs varied substantially between the different groups (see Additional file 3: Table S3). Among the *N. nucifera* groups, seed lotus showed the greatest number of insertions, tandem duplications, inversions and total and unique SVs, followed by wild sacred lotus. The number of each type of SV and unique SVs in flower lotus was slightly higher than in rhizome lotus. American lotus group displayed the most unique SVs among the five groups.

### Genetic relationships of wild *N. nucifera*

After the glacial period, two species of *Nelumbo* survived and spread from temperate to tropical areas. In this study, we found that lotus has maintained considerably high genetic diversity not only between the two species of lotus but also within a single species. The relatively safe water habitat of these plants, along with their ability to undergo both sexual and vegetative reproduction and the longevity of their seeds have probably contributed to the maintenance of a high level of genetic diversity in the lotus population. Some scholars believe that *N. nucifera* has two ecotypes: temperate lotus and tropical lotus [27]. Temperate lotus is distributed across the area north of 20° north latitude, where lotus plants show a significant annual growth cycle with different seasonal climate changes. Tropical lotus is distributed across the tropical area south of 17° north latitude and exhibits perennial growth. To resolve the genetic relationships of wild *N. nucifera*, we performed a population structure analysis using only the wild accessions (see Additional file 9: Figure S4). We found that the wild accessions could be divided into three geographically diverse groups in the neighbor-joining (NJ) tree, corresponding to northeast + midland + eastern China; Indonesia; and southern China + Thailand. The lotus accessions from tropical area (Thailand and Indonesia) did not cluster together, suggesting that the lotus divergence could begin with splitting tropical-subtropical Eurasian and American species followed by a rise of a common ancestor of the two peripheral temperate and Indonesian groups. Moreover, population structure analysis and principal component analysis (PCA) indicated that there might be abundant genetic variations among lotus plants from tropical areas. According to our observations, some tropical lotus sources introduced to Wuhan (Hubei Province, P.R.C.) show an annual growth cycle similar to that of temperate lotus. These findings indicate that the division of *N. nucifera* into temperate lotus and tropical lotus according to habitat is inappropriate. The differentiation of lotus within tropical areas and between tropical and temperate areas will require further research with more samples.

### Population structure of wild and cultivated lotus

**Fig. 2:**
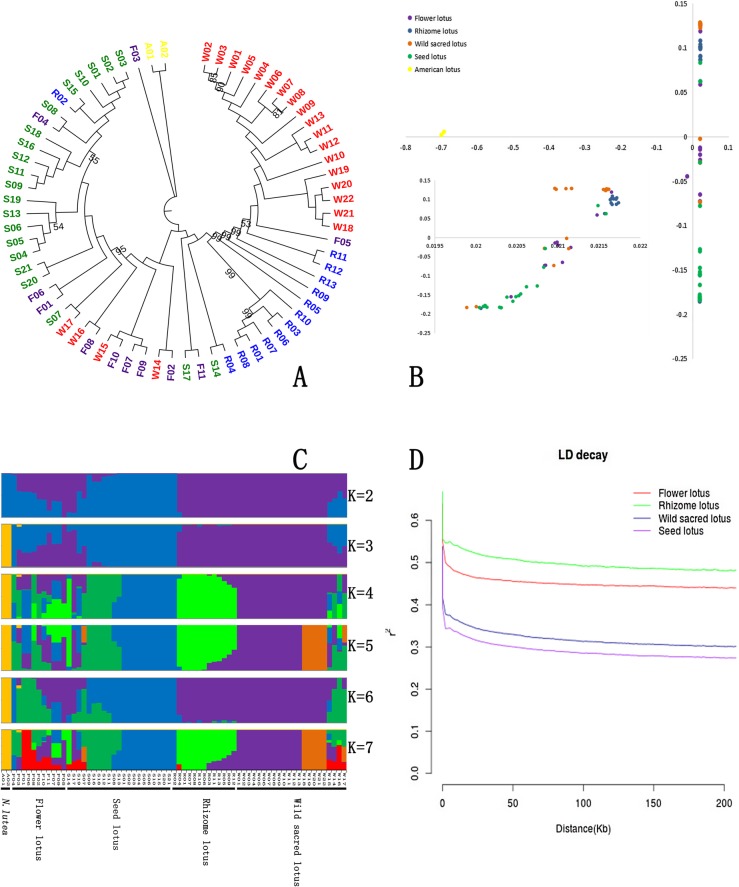
Population structure and LD decay in lotus. **a** The neighbor-joining tree of the 69 lotus accessions with bootstrap =1000 and the bootstrap values less than 100 were labelled. The accessions shown in red are wild sacred lotus, while yellow indicates American lotus, and purple, blue, and green represent flower lotus, rhizome lotus and seed lotus, respectively. **b** Principal component analysis (PCA) of the 69 lotus accessions. Two accessions of American lotus were from locations far from the sacred lotus accessions. The PCA of 67 accessions (*Nelumbo nucifera*) is shown on the left bottom side. **c** Population structure (k = 2–7) of the 69 lotus accessions determined by FRAPPE. Each accession is represented by a vertical bar, and the length of each colored segment in each vertical bar represents the proportion contributed by ancestral populations. **d** Differences in linkage disequilibrium (LD) between the wild and cultivated lotus groups. LD decay determined via squared correlations of allele frequencies (r2) against the distance between polymorphic sites in cultivated and wild lotus

On the basis of genetic distance, a neighbor-joining (NJ) tree was constructed (Fig. 2). The NJ tree contained two major clades, corresponding to the *N. nucifera* accessions and *N. lutea* accessions. It showed considerable genetic differentiation between the two species, which supports the findings of taxonomic studies. Among *N. nucifera* clade, seed lotus accessions and rhizome lotus accessions clustered together, respectively, obviously separating from the wild accessions. The clear genetic separation between the wild and cultivated groups (especially the rhizome lotus and seed lotus groups) confirmed the domestication event in lotus. Moreover, flower lotus accessions dispersed, suggesting their complex genetic background.

The results of principal component analysis (PCA) were consistent with the NJ tree (Fig. 2). Using the first and second eigenvectors, the 69 materials were divided into three groups: *N. lutea*; rhizome lotus + 18 wild sacred lotus accessions; and seed lotus + flower lotus + four wild sacred lotus accessions. Among the cultivated varieties, the rhizome lotus group exhibited a tight cluster, suggesting relatively low genetic variation. In contrast, seed lotus and flower lotus were more dispersed, indicating higher diversity than that of rhizome lotus. We also noted that the flower lotus groups were partly mixed with seed lotus and rhizome lotus, suggesting that flower lotus developed from the two populations and were derived for ornamental purposes. Insights might be obtained from the recorded domestication history of lotus [28–31]. Archaeological evidence and ancient books from China indicate the adoption of lotus as an ornamental plant and the use of its seeds and rhizomes as food. This domesticated population was probably the common ancestor of cultivated lotus. Lotus was first planted in a garden by King Fu Chai in 473 B.C., which marked the beginning of the domestication of flower lotus [30], after which the phenotypes of lotus were gradually differentiated into field lotus and garden lotus.

Although ancient Chinese people were digging and consuming lotus rhizomes 3000–5000 years ago [28], few records were found regarding rhizome lotus domestication. Based on the NJ tree and PCA results, rhizome lotus shows high genetic differentiation from seed lotus and flower lotus. Hence, the possibility that rhizome lotus was domesticated independently from different populations of flower lotus and seed lotus was considered. To further analyze the domestication history of lotus, we constructed a multilevel (K = 2, 3…7) population structure to estimate the maximum likelihood ancestry and the proportion of the ancestral property in each individual (Fig. 2). The minimum coefficient of variation(CV) error existed when k = 5, indicating it made most biological sense when k = 5. Rhizome lotus was separated from wild sacred lotus for K = 5, which supports the hypothesis that rhizome lotus was monophyletic and genetically quite homogeneous. Seed lotus showed two subgroups when K = 5, suggesting that there could be two types of seed lotus. Moreover, for K = 2, we found a division between rhizome lotus and seed lotus/flower lotus, and the flower lotus accessions showed evidence of admixture when K = 2, supporting the PCA analysis that flower lotus possibly domesticated from two ancestors. Meanwhile, a recent history of introgression from wild lotus in flower lotus as identified (K = 4–7).

Interestingly, a few of the accessions occurred at unexpected positions in both the PCA diagrams and NJ trees (Fig. 2). Although these accessions are treated as a certain cultivated type, they showed admixed genetic backgrounds, exhibiting phenotypes both from their own population and others (see Additional file 10: Figure S5). For example, sample F04 is a flower lotus accession with beautiful flowers, but its carpellary number is ≥24, which is equivalent to the average number for seed lotus accessions. These accessions are valuable resources for breeding multipurpose cultivars.

To estimate the linkage disequilibrium (LD) patterns in different lotus groups, we calculated r^2^ between pairs of SNPs. Linkage disequilibrium decayed to its halfmaximum(decaying to r2 of 0.75) at 620 bp, 510 bp, 1.37 kb, and 1.49 kb for wild sacred lotus, flower lotus, rhizome lotus, and seed lotus, respectively. The level of LD observed in lotus was much lower than that of other plants (*A. thaliana*: ~ 3 kb to 4 kb; soybean: ~ 75 kb to 150 kb; rice: ~ 10 kb to 200 kb; cucumber: ~ 3.2 kb to 140.5 kb; and cultivated sorghum: ~ 15 kb) [14, 17, 19, 32, 33]. The lower LD found in flower lotus among domesticated groups suggested the occurrence of frequent hybridization events during flower lotus domestication, compared with seed lotus and rhizome lotus. Such a level of LD in lotus groups is useful for studying population structure and association mapping.

### Regions (genes) under artificial selection

**Fig. 3:**
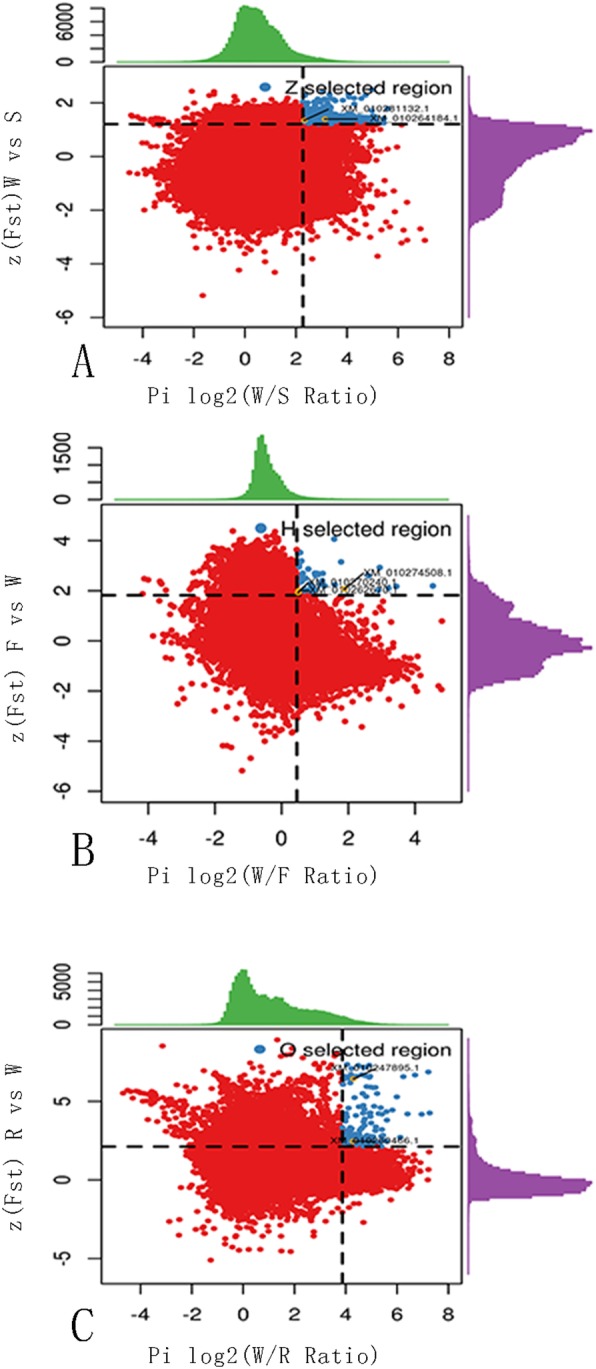
Distribution of log2π ratios and Z(F_ST_) values, calculated in 10-kb windows sliding in 5-kb steps. The selected genes disscussed in the main text were marked in the selected rigions (blue pionts). Data points located to the right of the right vertical dashed lines (corresponding to the 5% right tails of the log_2⁡π ratio distribution, where the log2π ratio is 2.27 in (**a**), 0.46 in (**b**), 3.87 in (**c**)) and above the horizontal dashed line (the 5% right tail of the Z(F_ST_) distribution, where Z(F_ST_) is 1.21 in (**a**), 1.82 in (**b**), 2.22 in (**c**)) were identified as selected regions for seed lotus, flower lotus and rhizome lotus(blue points), respectively

**Fig. 4:**
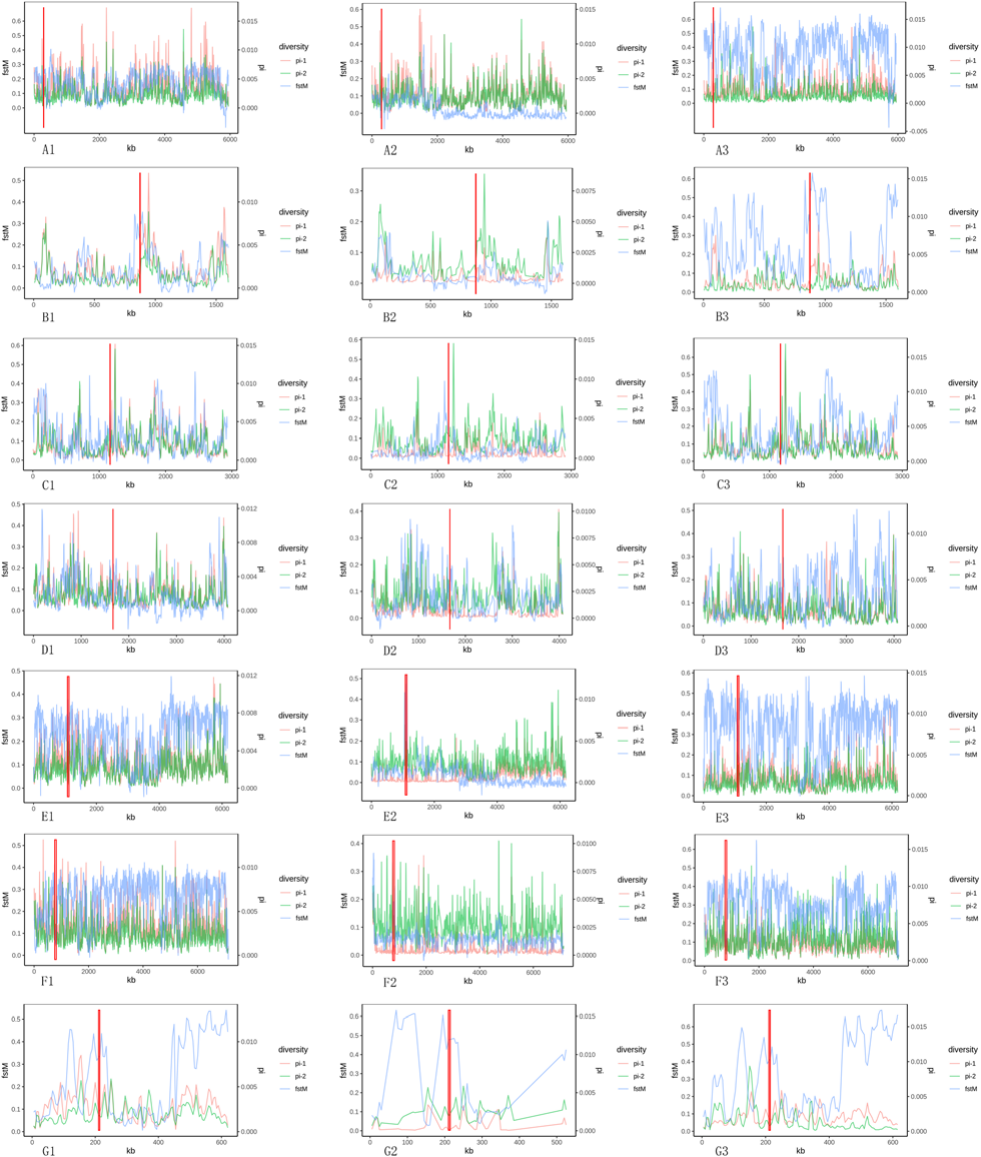
Examples of seven genes with strong signals of a selective sweep in cultivated lotus. Figure A, B, C, D, E, F and G correspond to the SUPERMAN-like gene(XM_010264184.1), legumin A-like gene(XM_010281131.1), 3-oxo-Δ 4,5-steroid 5β-reductase gene(XM_010274509.1), EFR gene(XM_010270240.1), UGT80B1 gene (XM_010262670.1), EMBRYONIC FLOWER 2-like gene (XM_010260462.1) and expansin-A13-like gene (XM_010247895.1) respectively. The abscissa of each figures are the physical distance. The left ordinate is the fstM value and the right ordinate is the pi value; pi-1, pi-2 and fstM values are presented in pink, green and blue and the red rectangles show the gene position. In the first column of the graph, the pi-1 and pi-2 stand for the pi values of the selected genes of flower lotus and wild lotus respectively; the pi-1 and pi-2 stand for the pi values of rhizome lotus and wild lotus respectively in the second column and the pi-1 and pi-2 stand for the pi values of wild lotus and seed lotus respectively in the third column

The divergence between the wild and cultivated lotus groups was significantly derived from three types of artificial selection. Flower pattern and color and other ornamental variations are the key phenotypic traits upon which flower lotus selection is based. The seed number per flower and the whole-field yield are the most important factors in seed lotus selection. For rhizome lotus selection, the morphology and yield of the underground stem are the characteristics of interest. Compared with wild accessions, regions that had undergone selection in the domesticated group displayed a low level of genetic diversity and presented skewed allele frequency spectra. Parameter analysis has been shown to be a reliable method for identifying putative artificially selected genes in domesticated species, including rice [14], maize [15], silkworms [25], cattle [34], and pigs [35]. In the present study, the combination of F_ST_(wild lotus/seed lotus, flower lotus/wild lotus and rhizome lotus/wild lotus) and θπ analyses was used to detect regions of selection in lotus (Fig. 3 and Fig. 4).

Compared with the whole-genome data for each cultivated group, polymorphism θπ values were significantly decreased in regions under selection, whereas the nonsynonymous/synonymous ratio was increased in CDS regions under selection (Table 2). Seed lotus exhibited the greatest number of selected regions and genes, followed by rhizome lotus. Flower lotus showed the lowest number of selected regions and genes. A total of 1214 selected regions were found in seed lotus, including 2176 selected genes, with 1.79 genes being located in each selected region on average. A total of 95 selected regions were found in rhizome lotus, including 77 selected genes, with 0.81 genes in each region on average. A total of 37 selected regions were identified in flower lotus, including 24 selected genes, with 0.65 genes in each region on average (Table 2, see Additional file 11: Dataset S1).

**Table 2:** Statistics of the regions under selection in lotus groups

Group	Number of snp	θ_π_ (10^−3^)	Nonsynonymous	Synonymous	Ratio of Nonsynonymous/Synonymous	regions under selection	Genes under the selection
Flower lotus	2094	0.000597	35	10	3.50	37	24
Seed lotus	188,960	0.000296	1771	1443	1.23	1214	2176
Rhizome lotus	7632	0.000134	54	24	2.25	95	77

The selected genes of seed lotus were mainly involved in the biological processes of starch synthesis, sugar transport, flowering regulation, flower differentiation and development, and seed development (see Additional file 11, S1.1 in Dataset S1, Additional file 12: Figure S6 and Additional file 13: Figure S7). The seed size and weight of crops increase during the domestication process [36]. Seed lotus displays typical domestication phenotypes, exhibiting larger and heavier seeds than of its wild ancestor. Recently, a SUPERMAN-like gene was identified in chrysanthemum that affects lateral bud outgrowth and flower organ development in tobacco, including enhancement of seed weight and size [37]. A SUPERMAN-like (XM_010264184.1) gene was also found among the selected genes of seed lotus (Fig. 3a and Fig. 4.A1,A2,A3). We hypothesize that this gene has played an important role in seed lotus domestication, affecting lotus seed weight and size in particular. Regarding nutritional quality, lotus seeds are an excellent source of food protein for humans, with a protein content exceeding 7.8% in fresh seeds, which is much higher than that of fresh chestnut (4.0%), water caltrop (3.6%), and ginkgo (6.4%) [38]. This quality has resulted from the artificial selection of seed lotus. For example, a legumin A-like gene (XM_010281131.1) was identified (Fig. 3a and Fig. 4.B1,B2,B3), which is involved in storage protein synthesis in seeds [39].

The flower lotus group currently includes more than 400 cultivars, including many ornamental phenotypes. Humans have apparently placed less selection pressure on the enhancement of ornamental characteristics in flower lotus. Thus, genes related to flower morphology present comparably high levels of variation to the selected genes. The selected genes were mainly enriched in the biological processes of translation and lipid glycosylation (see Additional file 11, S1.2 in Dataset S1, Additional file 14: Figure S8 and Additional file 15: Figure S9). Resistance genes have played an important role in the domestication of crops such as rice [14], corn [15], and cucumber [19], which have been subjected to significant pressure from artificial selection. The resistance genes identified among the selected genes provide classical evidence of flower lotus domestication. For example, one of the selected genes encodes 3-oxo-Δ 4,5-steroid 5β-reductase (XM_010274509.1), which is a key enzyme in the synthesis of cardiac glycoside [40](Fig. 3b and Fig. 4.C1,C2,C3). Cardiac glycoside is highly toxic to insects and mammals, thus enhancing insect resistance in flower lotus. The EFR (XM_010270240.1)(Fig. 3b and Fig. 4.D1,D2,D3) and UGT80B1 (XM_010262670.1) (Fig. 3b and Fig. 4 E1,E2,E3) genes are responsible for antibacterial immunity [41] and freezing and heat stress resistance [42], respectively.

When sexual reproduction is blocked in plants, there is complete transfer of resources from sexual to asexual reproduction, as verified by an artificial experiment in *Helianthus tuberosus* [43]. Rhizome lotus appears to provide a natural representative case of this phenomenon. Given the absence or scarcity of flowers in rhizome lotus plants, their rhizomes grow larger than those of other lotus types. Among the selected genes (see Additional file 11, S1.3 in Dataset S1, Additional file 16: Figure S10 and Additional file 17: Figure S11), two genes that significantly contribute to the typical characteristics of rhizome lotus were identified. One of these genes is the EMBRYONIC FLOWER 2-like gene (XM_010260462.1), encoding a polycomb group protein, which represses the reproductive development of *A. thaliana* [44] and may inhibit flowering in rhizome lotus as well (Fig. 3c and Fig. 4.F1,F2,F3). The other is the expansin-A13-like gene (XM_010247895.1), which belongs to the expansin family (Fig. 3c and Fig. 4.G1,G2,G3). The expansin family plays an important role in many biological activities, including fruit ripening, hypocotyl and coleoptile elongation, and leaf development [45–47]. In our previous report, the lotus α-expansin gene NnEXPA1 was shown to be an important factor in the final determination of rhizome size [48]. Therefore, this selected gene improves the size of rhizome lotus.

## Discussion

In contrast to conventional crops that rely on seed reproduction, lotus cultivars depend on vegetative reproduction. Unique genetic variation is effectively maintained in a specific cultivar. However, this method also reduces the motivation to develop new cultivars of lotus. For example, in the early Western Zhou Dynasty (1046 B.C.―771 B.C), lotus rhizome was one of the 40 vegetables recorded in China, which means that rhizome lotus domestication occurred 3000 years ago [28]. In the sixteenth century, a rhizome lotus with white flowers was listed in the famous Chinese Medicine book Ben Cao Gang Mu [49]. Current rhizome lotus cultivars mainly have white flowers and exhibit the same traits as rhizome lotus from 500 years ago. Although domestication has obviously significantly increased the heterozygosity and the level of genetic diversity in lotus cultivars compared to the wild lotus, among the three cultivated groups, flower lotus exhibited the greatest variation, followed by seed lotus and rhizome lotus showed the lowest variation. People prefer cross-breeding of flower lotus and seed lotus, rather than rhizome lotus. It seems minimal changes have been observed in rhizome lotus cultivars over this long time period. On the other hand, according to archaeological discoveries and history records domestication of three types of cultivated lotus began in the middle and downstream of the Yellow River and Yangtze River [50], which is a small area compared with the distribution area of the lotus. Thus, the low motivation for lotus breeding especially in rhizome lotus and the relatively narrow origin of cultivated lotus may negatively influence the sustainable development of the lotus industry. As the lotus cultivation area expands, current cultivars are subjected to different stresses, such as climate change and pathogen infection. So, the genetic diversity of lotus must be maximized to develop new or improved lotus cultivars. Wild lotus populations that are distant from current cultivars in terms of both geographic location and genetic background, such as those from northeast China, showing cold resistance, and tropical areas, showing a long flowering period, are a good choice for use in future lotus breeding.

During our field investigation, we found that the habitat of wild lotus faces serious threats from the increasing human population, urban development, and environmental pollution. Some wild lotus populations have disappeared from their previous habitat, and some have become smaller in size. Hence, we propose the establishment of protected areas for wild lotus in its traditional habitats and the development germplasm resource gardens, establishment of a seed bank for ex-situ conservation as soon as possible.

## Conclusions

The lotus is native to tropical Asia and Australia and is commonly cultivated in water gardens for show purposes also though primarily it is cultivated for its edible stems and seeds for thousands of years. It is amazing that the lotus plant is valuable for not only the physical well-being but even the spiritual health of humans. This study provides a large dataset showing the genome variation of lotus. A total of 74.99% of the detected SNPs were distributed across intergenic regions, and 1.89% of the detected SNPs were distributed across CDSs. The wild lotus population has maintained rich variation; meanwhile domestication increased the level of genetic diversity in cultivated lotus compared to the wild. Flower lotus showed the greatest variation, followed by seed lotus and then rhizome lotus. In this study a set of domestication genes were identified from three type of cultivated lotus, respectively. These genes include the gene encoding the protein that enhances insect resistance, improves seed weight and size, or regulates lotus rhizome size, which may be of agronomic importance to flower lotus, seed lotus and rhizome lotus, respectively. The identified SNPs and candidate selected genes during domestication provide a valuable resource for further research and for the improvement of lotus. Moreover, this study provides the initial steps toward a comprehensive genome-wide assessment of an aquatic crop and offers an important reference for other aquatic crops and plants.

## Methods

### Plant material and sequencing

All samples were grown in the greenhouses at the Wuhan University, Wuhan. Leaf tissues were collected, and DNA was extracted using a standard protocol [51]. The insert size of the libraries was 500 bp, and the paired-end reads were 125 bp. All libraries were sequenced using the high-throughput Illumina HiSeq TM 2000 instrument.

### Mapping and variation calling

Reads passing Illumina’s quality control filter were used for alignment to the reference and variation calling. For variation calling, we selected *N. nucifera* ‘China Antique’ as the reference genome (ftp://ftp.ncbi.nlm.nih.gov/genomes/all/GCA_000365185.2_Chinese_Lotus_1.1), and BWA (0.7.10) software [52] was used to map all the reads from each sample to the reference genome. SAMtools [53] was employed to convert the mapping results to bam format, and the results were further sorted. Heterozygosity was the quotient of the number of heterozygous SNPs divided by total chromosome length. The reads from PCR duplicates were removed with picard tools (picard-tools-1.119). Variation detection was performed with the Genome Analysis Toolkit (GATK, version 3.1) [54]. Through multisample analysis, we aligned all reads together against the reference genome, with a coverage of greater than twice and smaller than 1500 times. After adding headers for processing of the reads, realignment around indels was performed with the Realigner TargetCreator package to identify regions that needed to be realigned. Furthermore, IndelRealigner was used to perform realignment within these regions. Index files were generated by SAMtools, and the diversity was calculated by VCFtools [55]. We employed HaplotypeCaller to identify differences (SNPs, indels) in each variety. The threshold of SNP calling was set to 20 for both base quality and mapping quality, and the minor allele frequency threshold applied to the SNPs dataset was set to 0.1. All variations were joined together by GenotypeGVCFs.

### Validation of SNP calling

To evaluate the accuracy of SNP calling, we randomly selected 19 DNA regions containing 834 SNP loci for PCR amplification and Sanger sequencing.

### Phylogenetic analysis and population structure

Using the SNPs from all 69 varieties, a neighbor-joining tree was constructed using TreeBeST (version 1.9.2) with 1000 bootstrap replicates [56]. Population structure was investigated using FRAPPE (version 1.1) [57] and the optimal of K was calculated by admixture [58]. In addition, we performed PCA using GCTA(version 1.24.4) [59]. Two-dimensional coordinates were plotted for the 69 lotus accessions.

### Linkage disequilibrium analysis

To evaluate the level of LD in each pedigree, the correlation coefficients (r^2^) of the SNPs were calculated using Haploview software [60].

### Identification of candidate selective regions

A sliding-window method (10 kb sliding windows with a step of 5 kb) was used to calculate π ratios (θπ_cultivated/θπ_wild) and genetic differentiation (ZF_ST_) between the two populations [35]. To identify potential sweeps affected by artificial selection, we considered the distribution of log_2⁡π ratios and Z(F_ST_) values. We empirically selected DNA regions with both high log_(2)⁡π ratios (5% right tails of) and high Z(F_ST_) (5% right tails) as signals of selective regions across the genome, which should harbor genes that have undergone a selective sweep.

## Supplementary information

**Additional file 1: Table S1.** Summary of samples and sequencing.

**Additional file 2: Table S2.** Indels (insertions and deletions) summary.

**Additional file 3: Table S3.** SVs(Structure Variations) summary.

**Additional file 4: Table S4.** The accuracy rate of identified SNPs by PCR and Sanger sequencing.

**Additional file 5: Table S5.** Primer sequences for SNP validation by PCR amplification and Sanger sequencing.

**Additional file 6: Figure S1.** Geographic distribution of the 69 lotus accessions. The five different colors represent five different groups (red, wild sacred lotus; yellow, American lotus; pink, flower lotus; green, seed lotus; blue, rhizome lotus). This map was produced using Google Earth.

**Additional file 7: Figure S2.** Venn diagrams of the unique and common single-nucleotide polymorphisms (SNPs) in the five groups. The individual and overlapping areas in the Venn diagrams represent the number of unique or common SNPs among the lotus groups.

**Additional file 8: Figure. S3.** Venn diagrams of the unique and common short insertion and deletions (indels) in the five groups. The individual and overlapping areas in the Venn diagrams represent the number of unique or common indels among the lotus groups.

**Additional file 9: Figure S4.** Analysis of the phylogenetic relationships and population structure of wild sacred lotus. (A) Neighbor-joining phylogenetic tree constructed using SNP data. (B) Principal component analysis (PCA) of wild sacred lotus. (C) Bayesian clustering (STRUCTURE, K = 2-4) of wild sacred lotus.

**Additional file 10: Figure S5.** Morphology of the three lotus accessions based on multiple traits. Flower lotus accession F04 (A and B) exhibits both attractive flowers and many seeds per seedpod. Rhizome lotus accession R05 (C) exhibits both large rhizomes and many carpels per receptacle. Flower lotus accession F05 (D) exhibits both attractive flowers and swollen rhizomes. Bar indicates 10 cm.

**Additional file 11: Dataset S1.** The lists of all the genes under artificial selection in cultivated lotus groups.

**Additional file 12: Figure S6.** Gene Ontology analysis of the genes under artificial selection in seed lotus.

**Additional file 13: Figure S7.** Kyoto Encyclopedia of Genes and Genomes analysis of the genes under artificial selection in seed lotus.

**Additional file 14: Figure S8.** Gene Ontology analysis of the genes under artificial selection in flower lotus.

**Additional file 15: Figure S9.** Kyoto Encyclopedia of Genes and Genomes analysis of the genes under artificial selection in flower lotus.

**Additional file 16: Figure S10.** Gene Ontology analysis of the genes under artificial selection in rhizome lotus.

**Additional file 17: Figure S11.** Kyoto Encyclopedia of Genes and Genomes analysis of the genes under artificial selection in rhizome lotus.

**Additional file 18: Dataset S2.** The lists of the genes at 1% level as signals under artificial selection in cultivated lotus groups.

## Data Availability

All data generated or analysed during this study are included in this published article [and its supplementary information files]. The sequences reported in this paper have been deposited to NCBI Short Read Archive (accession no. SRP095218). The variants of indels and SNPs in VCF format have been deposited to Figshare (DOI 10.6084/m9.figshare.9969740 and 10.6084/m9.figshare.9974237).

## Abbreviations

CDS: Coding sequence
LD: Linkage disequilibrium
NJ: Neighbor-joining
P.R.C.: People Rpublic of China
PCA: Principal component analysis
PCR: Polymerase Chain Reaction
SNPs: Single-nucleotide polymorphisms
SVs: Structural variations
UTRs: Untranslated regions
