# Supplementary material for: Comparative population genomics reveals genetic divergence and selection in lotus, Nelumbo nucifera
Source: BMC Genomics. 2020 Feb 11;21:146. doi: 10.1186/s12864-019-6376-8 (PMC7014656; doi:10.1186/s12864-019-6376-8)
Supplement: Supplementary file 1 — Additional file 1: Table S1. Summary of samples and sequencing. [file 12864_2019_6376_MOESM1_ESM.docx]

**Table S1 Summary of samples and sequencing**

| **Number** | **Accession ID** | **Variety group** | **Local name** | **Species** | **Location** | **High-quality Base (bp)** | **Mapping Rate (%)** | **Duplication Rate(%)** | **Uniq Depth (X)** | **Uniq Coverage at least 1 × (%)** | **Het rate(%)** |
| --- | --- | --- | --- | --- | --- | --- | --- | --- | --- | --- | --- |
| F01 | H-1 | Flower lotus | Zhan Hong Tu | *N. nucifera* | Beijing, PRC | 11,142,179,500 | 86.21 | 4.2 | 12.86 | 98.55 | 0.45 |
| F02 | H-2 | Flower lotus | Zhuo Shang Lian | *N. nucifera* | Beijing, PRC | 9,563,055,500 | 87.01 | 3.59 | 11.01 | 99.08 | 0.49 |
| F03 | H-3 | Flower lotus | Hong Ri | *N. nucifera* | Hubei, PRC | 11,340,521,250 | 86.43 | 4.02 | 13.19 | 98.88 | 0.73 |
| F04 | H-4 | Flower lotus | Xia Ruo You Lang | *N. nucifera* | Japan | 10,877,081,250 | 86.39 | 4.11 | 12.56 | 97.86 | 0.26 |
| F05 | H-6 | Flower lotus | Juan Liu | *N. nucifera* | Hubei, PRC | 10,057,625,750 | 88.58 | 3.46 | 11.96 | 99.26 | 0.14 |
| F06 | H-7 | Flower lotus | Tang Zhao Ti Shi Lian | *N. nucifera* | Japan | 10,670,881,000 | 86.65 | 4.09 | 12.37 | 98.54 | 0.44 |
| F07 | H-8 | Flower lotus | Yin Du Jiao | *N. nucifera* | Hubei, PRC | 10,498,394,250 | 87.22 | 4.01 | 12.33 | 99.15 | 0.60 |
| F08 | H-9 | Flower lotus | Hong Tai Lian | *N. nucifera* | Hubei, PRC | 10,494,491,750 | 86.69 | 4.1 | 12.16 | 98.40 | 0.38 |
| F09 | H-10 | Flower lotus | Qian Ban Lian | *N. nucifera* | Yunnan, PRC | 9,298,768,250 | 87.26 | 3.84 | 10.98 | 99.01 | 0.58 |
| F10 | H-12 | Flower lotus | Da Sha Jin | *N. nucifera* | Yunnan, PRC | 11,868,853,250 | 86.92 | 4.73 | 13.91 | 99.24 | 0.64 |
| F11 | H-14 | Flower lotus | Tao Hong Ju | *N. nucifera* | Hubei, PRC | 9,760,060,750 | 87.19 | 4.05 | 11.42 | 98.83 | 0.21 |
| R01 | O-1 | Rhizome lotus | Liao Chen Ou Lian | *N. nucifera* | Shangdong, PRC | 12,568,541,000 | 87.41 | 4.32 | 14.88 | 99.13 | 0.20 |
| R02 | O-2 | Rhizome lotus | Piao Hua Lian | *N. nucifera* | Fujian, PRC | 12,012,309,250 | 86.19 | 4.34 | 13.88 | 97.61 | 0.14 |
| R03 | O-3 | Rhizome lotus | Hong Pao Zi | *N. nucifera* | Hubei, PRC | 11,217,433,000 | 88.19 | 3.84 | 13.23 | 99.08 | 0.13 |
| R04 | O-4 | Rhizome lotus | Mei Chuan Ou | *N. nucifera* | Hubei, PRC | 11,377,626,250 | 87.51 | 4.88 | 13.5 | 99.01 | 0.28 |
| R05 | O-5 | Rhizome lotus | Wu Zhi 2 | *N. nucifera* | Hubei, PRC | 10,578,774,250 | 88.17 | 3.67 | 12.5 | 99.18 | 0.19 |
| R06 | O-6 | Rhizome lotus | E Lian 8 | *N. nucifera* | Hubei, PRC | 11,364,016,250 | 88.08 | 3.82 | 13.37 | 99.14 | 0.15 |
| R07 | O-7 | Rhizome lotus | E Lian 6 | *N. nucifera* | Hubei, PRC | 10,951,116,500 | 88.10 | 3.69 | 12.87 | 99.09 | 0.18 |
| R08 | O-8 | Rhizome lotus | E Lian 5 | *N. nucifera* | Hubei, PRC | 10,512,851,250 | 87.77 | 3.6 | 12.33 | 99.10 | 0.18 |
| R09 | O-9 | Rhizome lotus | E Lian 4 | *N. nucifera* | Hubei, PRC | 11,181,679,250 | 88.21 | 3.84 | 13.27 | 99.24 | 0.18 |
| R10 | O-11 | Rhizome lotus | Zhen Zhu Ou | *N. nucifera* | Hubei, PRC | 12,477,640,500 | 87.48 | 4.24 | 14.69 | 98.98 | 0.15 |
| R11 | O-13 | Rhizome lotus | E Za 4 | *N. nucifera* | Hubei, PRC | 10,318,969,000 | 87.61 | 3.79 | 12.11 | 98.96 | 0.10 |
| R12 | O-14 | Rhizome lotus | Te Fen Ou | *N. nucifera* | Hubei, PRC | 10,111,620,000 | 87.73 | 3.69 | 11.86 | 99.14 | 0.16 |
| R13 | O-15 | Rhizome lotus | Dong He Zao Ou | *N. nucifera* | Zhejiang, PRC | 11,803,540,250 | 88.16 | 3.95 | 13.92 | 99.17 | 0.17 |
| S01 | Z-1 | Seed lotus | Cun San Lian | *N. nucifera* | Hunan, PRC | 11,201,078,000 | 86.26 | 4.14 | 13.01 | 97.80 | 0.21 |
| S02 | Z-2 | Seed lotus | Bai Xiang Lian | *N. nucifera* | Hunan, PRC | 9,587,843,000 | 86.11 | 4.56 | 11.06 | 97.54 | 0.21 |
| S03 | Z-3 | Seed lotus | Hong Xian Lian | *N. nucifera* | Hunan, PRC | 12,302,787,000 | 86.62 | 4.79 | 14.33 | 97.91 | 0.23 |
| S04 | Z-4 | Seed lotus | Tu Xuan Lian | *N. nucifera* | Zhejiang, PRC | 10,589,161,250 | 86.34 | 4.65 | 12.21 | 97.60 | 0.16 |
| S05 | Z-5 | Seed lotus | Chu Zhou Bai Lian | *N. nucifera* | Zhejiang, PRC | 10,626,032,500 | 86.24 | 4.68 | 12.19 | 97.34 | 0.13 |
| S06 | Z-6 | Seed lotus | Bai Hua Jian Lian | *N. nucifera* | Fujian, PRC | 10,304,353,750 | 86.82 | 3.63 | 11.83 | 97.57 | 0.13 |
| S07 | Z-7 | Seed lotus | Hong Hua Jian Lian | *N. nucifera* | Fujian, PRC | 11,914,067,250 | 87.05 | 4.66 | 13.99 | 98.96 | 0.54 |
| S08 | Z-8 | Seed lotus | Jian Xuan 17 | *N. nucifera* | Fujian, PRC | 12,474,402,500 | 86.80 | 4.59 | 14.57 | 97.98 | 0.27 |
| S09 | Z-10 | Seed lotus | Bai Ye Lian | *N. nucifera* | Jiangxi, PRC | 11,836,709,500 | 87.06 | 4.84 | 13.9 | 97.74 | 0.23 |
| S10 | Z-11 | Seed lotus | Gan Lian 62 | *N. nucifera* | Jiangxi, PRC | 12,121,404,500 | 86.68 | 4.69 | 14.19 | 97.77 | 0.23 |
| S11 | Z-12 | Seed lotus | Tai Kong Lian 36 | *N. nucifera* | Jiangxi, PRC | 11,544,013,750 | 87.14 | 4.61 | 13.58 | 97.89 | 0.28 |
| S12 | Z-13 | Seed lotus | Jing Guang 1 | *N. nucifera* | Jiangxi, PRC | 11,580,162,750 | 86.75 | 4.59 | 13.55 | 97.76 | 0.23 |
| S13 | Z-15 | Seed lotus | Jin Fu Rong 2 | *N. nucifera* | Zhejiang, PRC | 11,885,487,750 | 86.48 | 4.71 | 13.74 | 97.81 | 0.22 |
| S14 | Z-16 | Seed lotus | Wu Xi Lian | *N. nucifera* | Jiangsu, PRC | 11,625,031,750 | 87.50 | 4.62 | 13.75 | 99.04 | 0.29 |
| S15 | Z-17 | Seed lotus | Xuan Fu Rong 1 | *N. nucifera* | Zhejiang, PRC | 11,325,777,250 | 86.70 | 4.58 | 13.21 | 97.66 | 0.21 |
| S16 | Z-18 | Seed lotus | Fu Rong Lian | *N. nucifera* | Hunan, PRC | 11,439,024,000 | 86.54 | 4.57 | 13.27 | 98.01 | 0.32 |
| S17 | Z-19 | Seed lotus | Dong Gua Lian | *N. nucifera* | Fujian, PRC | 11,670,273,250 | 87.71 | 4.62 | 13.87 | 99.21 | 0.23 |
| S18 | Z-20 | Seed lotus | Xing Kong Mu Dan | *N. nucifera* | Jiangxi, PRC | 11,032,244,000 | 86.83 | 4.39 | 12.91 | 97.98 | 0.30 |
| S19 | Z-21 | Seed lotus | E Za 3 | *N. nucifera* | Hubei, PRC | 11,613,928,500 | 86.97 | 4.54 | 13.59 | 98.69 | 0.48 |
| S20 | Z-26 | Seed lotus | TZ 1 | *N. nucifera* | Jiangxi, PRC | 10,888,008,250 | 86.49 | 5.3 | 12.64 | 97.25 | 0.10 |
| S21 | Z-27 | Seed lotus | TZ 4 | *N. nucifera* | Jiangxi, PRC | 10,240,298,500 | 87.16 | 9.94 | 11.86 | 96.99 | 0.08 |
| W01 | Y-1 | Wild sacred lotus | Fang Zheng Lian | *N. nucifera* | Heilongjiang, PRC | 12,231,876,250 | 87.68 | 4.75 | 14.54 | 99.04 | 0.06 |
| W02 | Y-2 | Wild sacred lotus | Tong Jiang Lian | *N. nucifera* | Heilongjiang, PRC | 11,528,588,250 | 87.78 | 4.66 | 13.66 | 99.00 | 0.06 |
| W03 | Y-3 | Wild sacred lotus | Linkou lian | *N. nucifera* | Heilongjiang, PRC | 13,399,748,750 | 88.13 | 2.82 | 15.91 | 99.31 | 0.05 |
| W04 | Y-4 | Wild sacred lotus | Fangchuan lian | *N. nucifera* | Jilin, PRC | 11,764,418,500 | 88.28 | 2.80 | 13.81 | 99.24 | 0.05 |
| W05 | Y-5 | Wild sacred lotus | Xin Min Lian | *N. nucifera* | Liaoning, PRC | 10,470,050,750 | 88.46 | 4.26 | 12.38 | 99.01 | 0.06 |
| W06 | Y-6 | Wild sacred lotus | Bai Yang Dian Lian | *N. nucifera* | Hebei, PRC | 12,683,487,250 | 88.28 | 4.8 | 15.33 | 99.13 | 0.08 |
| W07 | Y-7 | Wild sacred lotus | Wei Shan Hu Lian | *N. nucifera* | Shandong, PRC | 11,423,394,500 | 87.37 | 4.89 | 13.48 | 98.96 | 0.08 |
| W08 | Y-8 | Wild sacred lotus | Pei Xian Lian | *N. nucifera* | Jiangsu, PRC | 11,032,098,000 | 87.66 | 4.95 | 13.1 | 98.91 | 0.07 |
| W09 | Y-9 | Wild sacred lotus | Hong Ze Hu Lian | *N. nucifera* | Jiangsu， PRC | 12,223,272,500 | 87.86 | 4.82 | 14.57 | 99.19 | 0.10 |
| W10 | Y-10 | Wild sacred lotus | Chao Hu Lian | *N. nucifera* | Anhui, PRC | 11,640,908,500 | 87.93 | 4.47 | 13.88 | 99.08 | 0.09 |
| W11 | Y-11 | Wild sacred lotus | Diao Cha Hu Lian | *N. nucifera* | Hubei, PRC | 11,494,114,750 | 87.91 | 4.41 | 13.75 | 99.02 | 0.08 |
| W12 | Y-12 | Wild sacred lotus | Hong Hu Lian | *N. nucifera* | Hubei, PRC | 12,872,026,000 | 87.70 | 4.84 | 15.35 | 99.15 | 0.08 |
| W13 | Y-13 | Wild sacred lotus | Dong Ting Hu Lian | *N. nucifera* | Hunan, PRC | 10,681,277,250 | 88.11 | 4.39 | 12.83 | 99.07 | 0.09 |
| W14 | Y-14 | Wild sacred lotus | Lian Hu Lian | *N. nucifera* | Jiangxi, PRC | 10,380,270,000 | 86.90 | 4.66 | 12.18 | 98.94 | 0.51 |
| W15 | Y-15 | Wild sacred lotus | Pu Zhe Hei Bai Lian | *N. nucifera* | Yunnan, PRC | 9,743,866,250 | 87.13 | 4.87 | 11.51 | 98.95 | 0.51 |
| W16 | Y-20 | Wild sacred lotus | Er Hai Lian | *N. nucifera* | Yunan， PRC | 12,021,395,750 | 87.34 | 4.59 | 14.18 | 98.49 | 0.36 |
| W17 | Y-17 | Wild sacred lotus | Tai Guo Lian 6 | *N. nucifera* | Chachoengsao Rayong, Thailand | 11,911,299,000 | 86.40 | 4.89 | 13.81 | 99.05 | 0.54 |
| W18 | Y-18 | Wild sacred lotus | Yin Ni Lian 1 | *N. nucifera* | Probolinggo, Indonesia | 13,061,132,000 | 87.79 | 4.17 | 15.26 | 98.59 | 0.07 |
| W19 | Y-22 | Wild sacred lotus | Yin Ni Lian 2 | *N. nucifera* | Bali，Indonesia | 11,884,163,000 | 87.09 | 3.78 | 13.77 | 98.70 | 0.06 |
| W20 | Y-23 | Wild sacred lotus | Yin Ni Lian 3 | *N. nucifera* | Kalimantan， Indonesia | 10,600,075,500 | 87.72 | 3.98 | 12.32 | 98.64 | 0.06 |
| W21 | Y-24 | Wild sacred lotus | Yin Ni Lian 4 | *N. nucifera* | Surabaya， Indonesia | 10,442,424,250 | 87.78 | 4.60 | 12.11 | 98.59 | 0.06 |
| W22 | Y-25 | Wild sacred lotus | Yin Ni Lian 5 | *N. nucifera* | Lamongan， Indonesia | 10,693,550,750 | 87.60 | 7.85 | 12.34 | 98.57 | 0.06 |
| A01 | Y-19 | American lotus | Mei Guo Lian 1 | *N.* *lutea* | USA | 11,432,370,000 | 84.06 | 4.91 | 11.91 | 87.91 | 0.25 |
| A02 | Y-21 | American lotus | Mei Guo Lian 2 | *N. lutea* | USA | 36,465,873,000 | 84.00 | 6.7 | 38.03 | 90.67 | 0.37 |
