# Supplementary material for: Comparative population genomics reveals genetic divergence and selection in lotus, Nelumbo nucifera
Source: BMC Genomics. 2020 Feb 11;21:146. doi: 10.1186/s12864-019-6376-8 (PMC7014656; doi:10.1186/s12864-019-6376-8)
Supplement: Supplementary file 2 — Additional file 2: Table S2. Indels (insertions and deletions) summary. [file 12864_2019_6376_MOESM2_ESM.docx]

**Table S2 Indels (insertions and deletions) summary**

| Group | n. | Total | Private | intergenic | intronic | UTR3 | UTR5 | UTR5;UTR3 | CDS | Gene numbers | Frameshift deletion | Frameshift insertion | Nonframeshift deletion | Nonframeshift insertion | Stopgain | Stoploss | Unknown |
| --- | --- | --- | --- | --- | --- | --- | --- | --- | --- | --- | --- | --- | --- | --- | --- | --- | --- |
| Flower lotus | 11 | 814526 | 24422 | 581239 | 212401 | 8478 | 6794 | 4 | 5610 | 2887 | 2176 | 1439 | 1023 | 682 | 90 | 13 | 187 |
| Seed lotus | 21 | 743383 | 62890 | 534072 | 190700 | 7237 | 6024 | 3 | 5347 | 2735 | 2078 | 1398 | 975 | 622 | 89 | 12 | 173 |
| Rhizome lotus | 13 | 572730 | 11207 | 408813 | 149258 | 5777 | 4771 | 3 | 4108 | 2195 | 1605 | 1085 | 702 | 500 | 71 | 9 | 136 |
| Wild sacred lotus | 22 | 712794 | 76034 | 509653 | 184649 | 7212 | 5862 | 5 | 5413 | 2846 | 2128 | 1359 | 1034 | 604 | 94 | 14 | 180 |
| American lotus | 2 | 2017540 | 1672002 | 1454144 | 510020 | 22860 | 17930 | 18 | 12568 | 5963 | 4753 | 3188 | 2317 | 1676 | 231 | 26 | 377 |
| Total | 69 | 2753718 | - | 1995912 | 687414 | 29094 | 23230 | 22 | 18046 | 7471 | 7032 | 4560 | 3420 | 2137 | 327 | 39 | 531 |
