# Supplementary material for: Comparative population genomics reveals genetic divergence and selection in lotus, Nelumbo nucifera
Source: BMC Genomics. 2020 Feb 11;21:146. doi: 10.1186/s12864-019-6376-8 (PMC7014656; doi:10.1186/s12864-019-6376-8)
Supplement: Supplementary file 3 — Additional file 3: Table S3. SVs(Structure Variations) summary. [file 12864_2019_6376_MOESM3_ESM.docx]

**Table S3 SVs(Structure Variations) summary**

| Groups | Flower lotus | Rhizome lotus | Seed lotus | Wild sacred lotus | American lotus | All SVs |
| --- | --- | --- | --- | --- | --- | --- |
| Group capacity (n) | 11 | 13 | 21 | 22 | 2 | 69 |
| Deletion | 195,292 | 155,214 | 237,579 | 207,987 | 217,365 | 518,454 |
| Deletion unique | 11,744 | 6,818 | 41,020 | 40,398 | 199,263 | - |
| Insertion | 115,246 | 97,586 | 136,926 | 117,938 | 113,529 | 259,970 |
| Insertion unique | 4,670 | 3,463 | 20,515 | 15,578 | 88,644 | - |
| Tandem Duplication | 10,608 | 10,025 | 15,360 | 12,967 | 5,768 | 27,036 |
| Tandem Duplication unique | 1,034 | 971 | 4,224 | 2,935 | 5,046 | - |
| Inversions | 3,472 | 3,265 | 6,607 | 4,815 | 1,851 | 13,044 |
| Inversions unique | 953 | 1,019 | 3,365 | 2,087 | 1,695 | - |
| Total | 324,618 | 266,090 | 396,472 | 343,707 | 338,513 | 818,504 |
| Unique in each group | 18,401 | 12,271 | 69,124 | 60,998 | 294,648 | - |
