# Supplementary material for: Comparative population genomics reveals genetic divergence and selection in lotus, Nelumbo nucifera
Source: BMC Genomics. 2020 Feb 11;21:146. doi: 10.1186/s12864-019-6376-8 (PMC7014656; doi:10.1186/s12864-019-6376-8)
Supplement: Supplementary file 4 — Additional file 4: Table S4. The accuracy rate of identified SNPs by PCR and Sanger sequencing. [file 12864_2019_6376_MOESM4_ESM.docx]

**Table S4 The accuracy rate of identified SNPs by PCR and Sanger sequencing**

| Sample | Group | Total test SNPs | Exact genotype | True SNPs (%) |
| --- | --- | --- | --- | --- |
| S19 | Seed lotus | 572 | 557 | 97.38% |
| F09 | Flower lotus | 561 | 554 | 98.75% |
| R10 | Rhizome lotus | 573 | 571 | 99.65% |
| W11 | Wild sacred lotus | 736 | 734 | 99.73% |
