# Supplementary material for: Comparative population genomics reveals genetic divergence and selection in lotus, Nelumbo nucifera
Source: BMC Genomics. 2020 Feb 11;21:146. doi: 10.1186/s12864-019-6376-8 (PMC7014656; doi:10.1186/s12864-019-6376-8)
Supplement: Supplementary file 5 — Additional file 5: Table S5. Primer sequences for SNP validation by PCR amplification and Sanger sequencing. [file 12864_2019_6376_MOESM5_ESM.docx]

**Table S5 Primer sequences for SNP validation by PCR amplification and Sanger sequencing**

| Number | ID | Forward primer (5'-3') | Reverse primer (5'-3') | Tm(°C) | Product size (bp) |
| --- | --- | --- | --- | --- | --- |
| L-1 | gi\|480357867\|gb\|KB846784.1\|_8858717 | GGTTGGCATAGTTAGGCATGA | AAGATCCGTTGAGCCTTCA | 56 | 698 |
| L-2 | gi\|480357866\|gb\|KB846785.1\|_8041420 | TGAGTGAAGCCTTGCACAAC | CGCTTGGAGGAGAATGACTC | 56 | 661 |
| L-3 | gi\|480357866\|gb\|KB846785.1\|_11852999 | TCGTGATGGATGGACCTAGA | AAGCTGACCTTGCAGTTGCT | 56 | 700 |
| L-4 | gi\|480357865\|gb\|KB846786.1\|_9888954 | TCACTTACGACACCGAAGCA | CAAGTCGATTGCCACGTGTA | 56 | 730 |
| L-5 | gi\|480357864\|gb\|KB846787.1\|_2196618 | CGATGCCACGCTAATAACTG | CGGTTGAATCATTCGAGTCC | 56 | 673 |
| L-6 | gi\|480357864\|gb\|KB846787.1\|_4579505 | GAAGGTGAGATGGCGCTAAG | TGCTCCTGTCAGAGACAAGGT | 56 | 713 |
| L-7 | gi\|480357862\|gb\|KB846789.1\|_3993507 | CAATGAACCGTTGGCCTAGA | GCAACGATCACTCCATCTCC | 56 | 685 |
| L-8 | gi\|480357862\|gb\|KB846789.1\|_5326587 | CTCTCTCTCCAGCTGGCATC | GCTCTTGCTGAGTGCTGAGT | 56 | 651 |
| L-9 | gi\|480357862\|gb\|KB846789.1\|_5849903 | CGAGCTTGAATGCTTGTGAA | GGCGCAGATGTGAACTCATT | 56 | 714 |
| L-10 | gi\|480357862\|gb\|KB846789.1\|_7573217 | TGTCAGCTCGCATCATTCTT | CATAGCACTTCGAACCACGA | 56 | 735 |
| L-11 | gi\|480357861\|gb\|KB846790.1\|_4783765 | TGAGGTCGCTCTGATGCTTA | TGCTGATGCAGGTGAAGAGT | 56 | 727 |
| L-12 | gi\|480357860\|gb\|KB846791.1\|_8695160 | CGAACCATTGCCAAGTACAA | CACCAGCAACAATGACGTTC | 56 | 742 |
| L-13 | gi\|480357858\|gb\|KB846793.1\|_8142439 | ACGAACCGAACTGACGGTTA | CTGGTGGAAGGCCAATACAT | 56 | 657 |
| L-14 | gi\|480357857\|gb\|KB846794.1\|_811449 | GCGATCTGGTCACTCCTAGC | CAGATGAACCACTCGTCGAA | 56 | 741 |
| L-15 | gi\|480357857\|gb\|KB846794.1\|_3484886 | AGTTCTCGCAGGTTCCTCAG | AGCTCCTTGGTGGTGAGCTA | 56 | 697 |
| L-16 | gi\|480357857\|gb\|KB846794.1\|_3778915 | CCACGCACATGTAAGACTCG | AGAACAGACCTCGCGTGAAC | 56 | 685 |
| L-17 | gi\|480357856\|gb\|KB846795.1\|_7391885 | GTGCGGTGACAAGTCACAAT | TCATCGGTACCTTCGTCCTC | 56 | 676 |
| L-18 | gi\|480357854\|gb\|KB846797.1\|_4714532 | TCCAATTACCGGTTAGAACCAT | CGGAAGTTGGCTCACGTATC | 56 | 737 |
| L-19 | gi\|480357853\|gb\|KB846798.1\|_921758 | AGTCCTACTCCTCGGCGAAT | AGGAGAGATCAACGGTGCAG | 56 | 668 |
| L-20 | gi\|480357853\|gb\|KB846798.1\|_2673057 | CCTCGGTTCACTCTACGACA | TGGCTCATCGCTAACCTGAT | 56 | 693 |
| L-21 | gi\|480357853\|gb\|KB846798.1\|_5317514 | CTAGGCGGCAGAAGAGACAC | CAGTTGCTGAGCTCAACTCG | 56 | 661 |
| L-22 | gi\|480357852\|gb\|KB846799.1\|_2191427 | GTGTTGCTCACCAGCATTCA | TGATGCCAGTTGACTTGAGC | 56 | 683 |
| L-23 | gi\|480357852\|gb\|KB846799.1\|_4026361 | GTCGGAAGAGTCTGCTCCAG | CTCTTCACCTTGCATGTCTCC | 56 | 677 |
| L-24 | gi\|480357852\|gb\|KB846799.1\|_4498747 | CAAGAAGCCTATAATTCGTTCTTCC | CGACATGGTTGCTTATGTGAA | 56 | 743 |
| L-25 | gi\|480357852\|gb\|KB846799.1\|_5227326 | AATAAGACAGGTCGGCAAGG | CCACACCGAAGTCTGTAGGAA | 56 | 651 |
| L-26 | gi\|480357851\|gb\|KB846800.1\|_1376946 | GCATGCCATAGCTTATGTTGC | AGGTCTGACATAACGGCTCAA | 56 | 696 |
| L-27 | gi\|480357851\|gb\|KB846800.1\|_2358618 | ATCTTGGCGTTCACGTCATT | TTGGACCTACATCAGGCCTATAA | 56 | 721 |
| L-28 | gi\|480357850\|gb\|KB846801.1\|_1241127 | GAAGTCCTTGCACGTCGTC | GGTCCTTAGGAGGTGGTTGA | 56 | 658 |
| L-29 | gi\|480357850\|gb\|KB846801.1\|_1609187 | GCACCTTGGTTCACAGGAAT | ATGTTGCATGGACTCGGACT | 56 | 664 |
| L-30 | gi\|480357850\|gb\|KB846801.1\|_5522740 | AGGCCACATAATCTGCTCCA | CAATGGCGGATCCAAGAC | 56 | 677 |
| L-31 | gi\|480357848\|gb\|KB846803.1\|_1407716 | GGAGATTCTGATGAGCATTGG | AACACCACAGTGAACTCATCTCA | 56 | 736 |
| L-32 | gi\|480357848\|gb\|KB846803.1\|_6170158 | GTTCGACCGAACCATGAGAT | AAGTGTGAGTCAGGCCGTGT | 56 | 737 |
| L-33 | gi\|480357847\|gb\|KB846804.1\|_4861036 | GTCCATCGGAGAGAGTGCAT | ACTAACCGCCATCCAGGTC | 56 | 664 |
| L-34 | gi\|480357847\|gb\|KB846804.1\|_6695324 | TTCACAAGTGCACAGCATGA | TTGAAGCTCCGAGCAATATG | 56 | 682 |
| L-35 | gi\|480357845\|gb\|KB846806.1\|_1881246 | GTTGCTCGGTTGTGAGCAT | CGAAGGAGAAGTCCAGATCG | 56 | 694 |
| L-36 | gi\|480357844\|gb\|KB846807.1\|_1521255 | GGCCACTGGAGGAATCTCTA | GGCCTCTCTCTTCTAAGGAGGT | 56 | 659 |
| L-37 | gi\|480357844\|gb\|KB846807.1\|_5036065 | TTGCTCTTCACCACTCCATT | GGTGAGCATTCATTGGCTCT | 56 | 656 |
| L-38 | gi\|480357842\|gb\|KB846809.1\|_3936510 | GGCTAGCGATTGACTGGTGT | GATGGCATCCAATCCAATTC | 56 | 720 |
| L-39 | gi\|480357842\|gb\|KB846809.1\|_4609211 | TTGCTGAAGGTGATCTTATGGA | ATTGGTGGTAAGGCAAGGTG | 56 | 663 |
| L-40 | gi\|480357841\|gb\|KB846810.1\|_2621509 | CCGATGGCTTACATGTGGTA | TCATACAGATAAGACCACACTCCA | 56 | 663 |
| L-41 | gi\|480357841\|gb\|KB846810.1\|_4607453 | AGCAGCGGACTACCAACAGT | TGTGCCACTTGAACTCCTTG | 56 | 650 |
| L-42 | gi\|480357839\|gb\|KB846812.1\|_4289628 | GGTATGTGTTCCATGTGGATG | TGAGCCACTATCATTCCTTCG | 56 | 711 |
| L-43 | gi\|480357837\|gb\|KB846814.1\|_998258 | CGCTGGTCATCCAAGTAGAAG | CCGCTGTTGTTCGTGTGTAT | 56 | 652 |
| L-44 | gi\|480357836\|gb\|KB846815.1\|_2993331 | TTGGACAGTTGAACCGTTGAT | GAGCAGACCGGACTTGATCT | 56 | 650 |
| L-45 | gi\|480357836\|gb\|KB846815.1\|_3216089 | CCGTGACGAGATCCTATTCG | CTCTTGTGGCCAGAGAGCTT | 56 | 720 |
| L-46 | gi\|480357834\|gb\|KB846817.1\|_969918 | GATCGCTTAGTCAGGCCAGT | TGACTATCCGGCCATTCAG | 56 | 736 |
| L-47 | gi\|480357834\|gb\|KB846817.1\|_1578464 | ATGGATAATACATCCACGGCTA | GATAGATACAAGCAATGTCGTCGT | 56 | 650 |
| L-48 | gi\|480357834\|gb\|KB846817.1\|_1649495 | CTCGGACTCTTGCCTGATGT | CAGAGAGGCCTATTGCAGAA | 56 | 711 |
| L-49 | gi\|480357833\|gb\|KB846818.1\|_2395607 | CGGAGTTGCTTCTCCTCCTT | GAGGTGGTGGTACGTCTGGT | 56 | 742 |
| L-50 | gi\|480357833\|gb\|KB846818.1\|_5095454 | CATCAGAATATCCAAGTCCTCCA | GCGATCACACGAATTGGAA | 56 | 749 |
| L-51 | gi\|480357832\|gb\|KB846819.1\|_2645730 | AAGGTGATGAGGTCGGTGTC | GCTCCATCAGCTCGTAGGAC | 56 | 666 |
| L-52 | gi\|480357831\|gb\|KB846820.1\|_599096 | TGGTTCCGTTCCTTAGTTGG | TGCCAACGCTATTACGACCT | 56 | 702 |
| L-53 | gi\|480357830\|gb\|KB846821.1\|_1126795 | AGGCACCTCGATCTCTGTTG | CGAGTATGTACTCCGCGACA | 56 | 653 |
| L-54 | gi\|480357830\|gb\|KB846821.1\|_4607176 | GCATATCGAGGAACCACCTC | ACAGGAAGGATGACGCAAGT | 56 | 737 |
| L-55 | gi\|480357828\|gb\|KB846823.1\|_3993101 | GGACTTGGCAAGGTTACTATTAGA | ACGGCCAAGTGAAGTGGAT | 56 | 664 |
| L-56 | gi\|480357826\|gb\|KB846825.1\|_3793604 | GGCATCCTTGATCTGTTGCT | TTGACACGGAGCAGTGGTT | 56 | 713 |
| L-57 | gi\|480357825\|gb\|KB846826.1\|_2439429 | TTCCGTGTCTTCCATTATGC | AATGCGACGTTCATTGACTG | 56 | 651 |
| L-58 | gi\|480357822\|gb\|KB846829.1\|_1028851 | CCACCAATCTGCTGTGTTCT | TCCTAGGATCCATGATGCAAT | 56 | 717 |
| L-59 | gi\|480357822\|gb\|KB846829.1\|_1557984 | AGAGGATGCAGAGGAAGACG | ATACACACGCGGATGTCAGA | 56 | 702 |
| L-60 | gi\|480357821\|gb\|KB846830.1\|_3745171 | TGCTCACCGACAAGTGACAT | GGCAGTTCGTTAGCAACCTC | 56 | 659 |
| L-61 | gi\|480357820\|gb\|KB846831.1\|_226937 | AAGTGGAGGTGAGTAGAATCCTG | AGATGCGCCTGATTGGATAC | 56 | 722 |
| L-62 | gi\|480357819\|gb\|KB846832.1\|_1455786 | TGGATATGACTCGACGCAAG | CCTCACTCCTAGCTGGCAAC | 56 | 688 |
| L-63 | gi\|480357818\|gb\|KB846833.1\|_1099268 | CAATCACAACAGGCAAGAACA | CCATTGCCAAGACCAAGAAT | 56 | 658 |
| L-64 | gi\|480357818\|gb\|KB846833.1\|_2706163 | TGTGGCCTCACATAGACCAA | GCGACACTACCTTACGAGTGC | 56 | 663 |
| L-65 | gi\|480357810\|gb\|KB846841.1\|_3224469 | TGATGAGCAATTGGTATTGG | CTACACCACCACCTCAACCA | 56 | 673 |
| L-66 | gi\|480357807\|gb\|KB846844.1\|_2263526 | CGAGCATAGGCGAAGCTATT | AGACCGCATCCTTGCTCTT | 56 | 700 |
| L-67 | gi\|480357807\|gb\|KB846844.1\|_2266754 | AGGACGTACCGAGCTGATTG | GAATGCACGTTCATCACGTT | 56 | 695 |
| L-68 | gi\|480357798\|gb\|KB846853.1\|_2001398 | ACCTTCATTCCTTCCACAGC | GCAAGAGGACTTCGACTGCT | 56 | 719 |
| L-69 | gi\|480357794\|gb\|KB846857.1\|_518812 | GGTGAGCCACAAGGTAATCG | CCATTGCGGCTATAAGCTCT | 56 | 656 |
| L-70 | gi\|480357791\|gb\|KB846860.1\|_1834119 | CCAACCTCATGGATGTAATGG | TGATTGACGTGCATGGATTA | 56 | 700 |
| L-71 | gi\|480357790\|gb\|KB846861.1\|_283607 | CGTCAAGTTAGTGACATGGTGAG | CCATCATCATCAGCCTTAACC | 56 | 689 |
| L-72 | gi\|480357790\|gb\|KB846861.1\|_2881662 | TGACAACATGAAGCACAGCA | CGCCACCACAATGTAGTTGTA | 56 | 735 |
| L-73 | gi\|480357779\|gb\|KB846872.1\|_2052428 | CAGCGGATTGCTTAAGAACG | CCTGTTAGTGATAATAGTGGCATGT | 56 | 704 |
| L-74 | gi\|480357778\|gb\|KB846873.1\|_2413236 | TGGTCTGTGGATGGTAAGCA | TAGCCAAGTGCCATGGTATG | 56 | 695 |
| L-75 | gi\|480357777\|gb\|KB846874.1\|_2375466 | TGAAGGCTAGTAGTGGTGCCTA | GCGTCAATGCGTCATGATAA | 56 | 690 |
| L-76 | gi\|480357776\|gb\|KB846875.1\|_858943 | CCTTCCGTTGTGTAGCCTATAA | CATGCGAGTGTATGGAATGAA | 56 | 682 |
| L-77 | gi\|480357773\|gb\|KB846878.1\|_877144 | TGATGGACGATCGATTAGAGG | CCGATCAGACACCATCACAT | 56 | 723 |
| L-78 | gi\|480357765\|gb\|KB846886.1\|_1808613 | CCACCGAAGCTCAGTCAGTT | GCCAAGCTAGATGGTTCTGC | 56 | 728 |
| L-79 | gi\|480357762\|gb\|KB846889.1\|_1437982 | CAAGACCAACACTTACGAAGTCC | CCTTATTGCCTAGGCGTCAC | 56 | 650 |
| L-80 | gi\|480357757\|gb\|KB846894.1\|_372589 | CTTGCACAATTCAGTTGGTGA | TGAGCCAGTATCCAATCAGACA | 56 | 708 |
| L-81 | gi\|480357756\|gb\|KB846895.1\|_1079841 | GGAATGGTGCCAAGATATGG | GCTCGGCATTCTATCCTTGA | 56 | 670 |
| L-82 | gi\|480357756\|gb\|KB846895.1\|_1594546 | GAGAGAGGCAATGAATGAAGAA | AAGGAAGTATCTGAGATGGCCTAA | 56 | 679 |
| L-83 | gi\|480357755\|gb\|KB846896.1\|_177469 | CTTCCAAGTTCCATCCATGC | ATTCATTGCACCGAATCAAC | 56 | 726 |
| L-84 | gi\|480357755\|gb\|KB846896.1\|_965951 | GGATTCAGCTCTATGGCTATCA | TTGGAGTAAGGTGTGCAAGC | 56 | 700 |
| L-85 | gi\|480357755\|gb\|KB846896.1\|_1141068 | CGAGCCATTAATTGGCAGAA | GCCTTAGAGGCTTGTTGGTG | 56 | 664 |
| L-86 | gi\|480357755\|gb\|KB846896.1\|_1647524 | TAGCAGGTTAGGTCGGCAGT | CCATTCAGAGCCTTATCTGTCC | 56 | 709 |
| L-87 | gi\|480357755\|gb\|KB846896.1\|_1764067 | CCTCGGAGGTAGTTGTGCAT | GGTAGATGTCCTCGGAGTGC | 56 | 688 |
| L-88 | gi\|480357751\|gb\|KB846900.1\|_446905 | CCAAGCTGAAGCCTCGTT | CCAAGAGTTCCTAGATTGTTCTCC | 56 | 678 |
| L-89 | gi\|480357742\|gb\|KB846909.1\|_1557895 | CGTCGTCATCCAACGAATC | TGGCTTGAGCAATAGCATTATC | 56 | 674 |
| L-90 | gi\|480357739\|gb\|KB846912.1\|_592900 | TCTTGGTTGTGGTGGAACAA | GGTTGACGTTGGTGAGCAAT | 56 | 731 |
| L-91 | gi\|480357737\|gb\|KB846914.1\|_340728 | CAGTCCGTATAGGTGCACAGG | GAAGACCAAGAGTGCCGAAC | 56 | 723 |
| L-92 | gi\|480357733\|gb\|KB846918.1\|_1545287 | AGCTGCCAGAATCGATGAGT | GGAATCAATTGGCACCATCT | 56 | 681 |
| L-93 | gi\|480357731\|gb\|KB846920.1\|_745637 | TTGCAGCCACACCAAGATTA | TTGTGACTGTTCAAGGTGTCTTC | 56 | 739 |
| L-94 | gi\|480357723\|gb\|KB846928.1\|_520717 | TTGGCCTTAGGCATTCAATC | TTGCATCAGATGAGCGTGTA | 56 | 741 |
| L-95 | gi\|480357722\|gb\|KB846929.1\|_1369164 | CAAGCTATGGCAAGCCAAC | TTAGCTTCTGAATGCGGACA | 56 | 676 |
| L-96 | gi\|480357718\|gb\|KB846933.1\|_1369191 | TTGGATTGCATGAACTAACACC | CGGATCCTCCAGGTCTTGTA | 56 | 661 |
| L-97 | gi\|480357717\|gb\|KB846934.1\|_268172 | CAATCCGACACCTAGTGCAG | GGAGCAGTACGCCATGATCT | 56 | 692 |
| L-98 | gi\|480357715\|gb\|KB846936.1\|_896458 | CATGGTGATGTCATGGCTGT | TCCTCTGACCTTGCTGTCCT | 56 | 683 |
| L-99 | gi\|480357711\|gb\|KB846940.1\|_443454 | CGGAGCAACTCAATGATACG | TTGCTTCCGCCTGTAAGTCT | 56 | 694 |
| L-100 | gi\|480357707\|gb\|KB846944.1\|_415064 | AGGTCCATTAAGGCATCAGC | CCACCATTCCTCCACCAA | 56 | 689 |
