# Supplementary figures and images for: Comparative population genomics reveals genetic divergence and selection in lotus, Nelumbo nucifera
Source: BMC Genomics. 2020 Feb 11;21:146. doi: 10.1186/s12864-019-6376-8 (PMC7014656; doi:10.1186/s12864-019-6376-8)

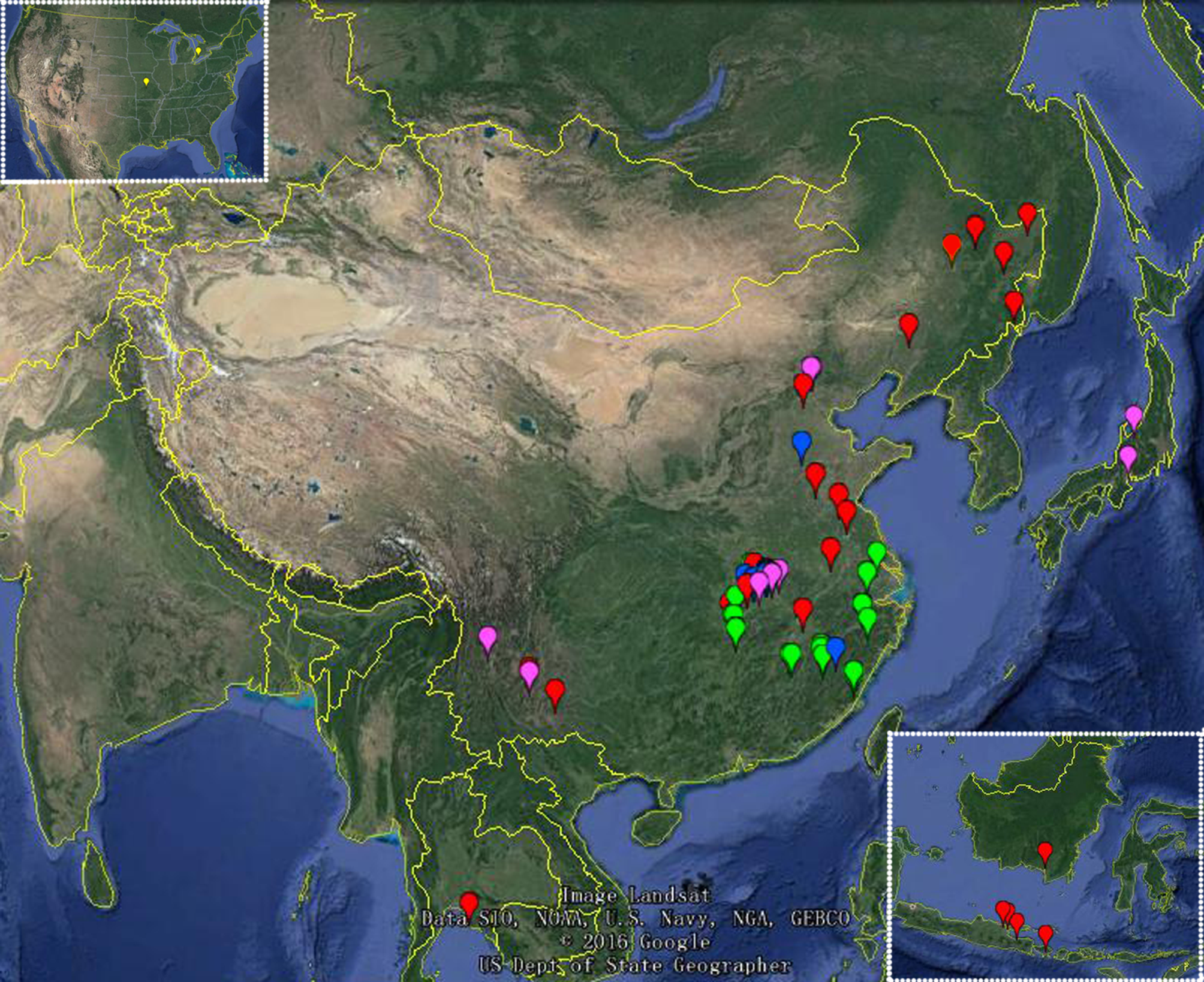

Supplement: Supplementary file 6 — Additional file 6: Figure S1. Geographic distribution of the 69 lotus accessions. The five different colors represent five different groups (red, wild sacred lotus; yellow, American lotus; pink, flower lotus; green, seed lotus; blue, rhizome lotus). This map was produced using Google Earth. [file 12864_2019_6376_MOESM6_ESM.tif]

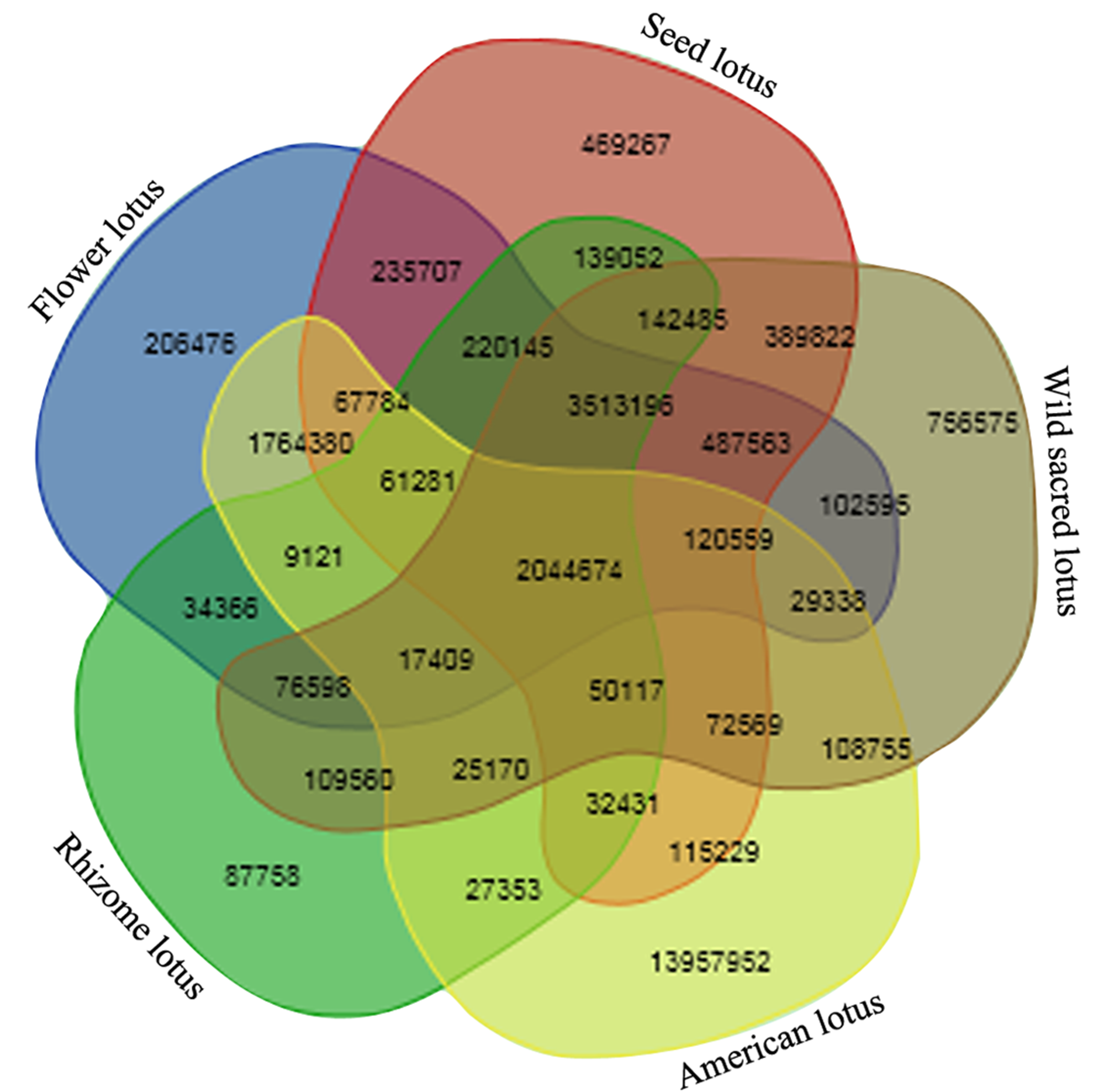

Supplement: Supplementary file 7 — Additional file 7: Figure S2. Venn diagrams of the unique and common single-nucleotide polymorphisms (SNPs) in the five groups. The individual and overlapping areas in the Venn diagrams represent the number of unique or common SNPs among the lotus groups. [file 12864_2019_6376_MOESM7_ESM.tif]

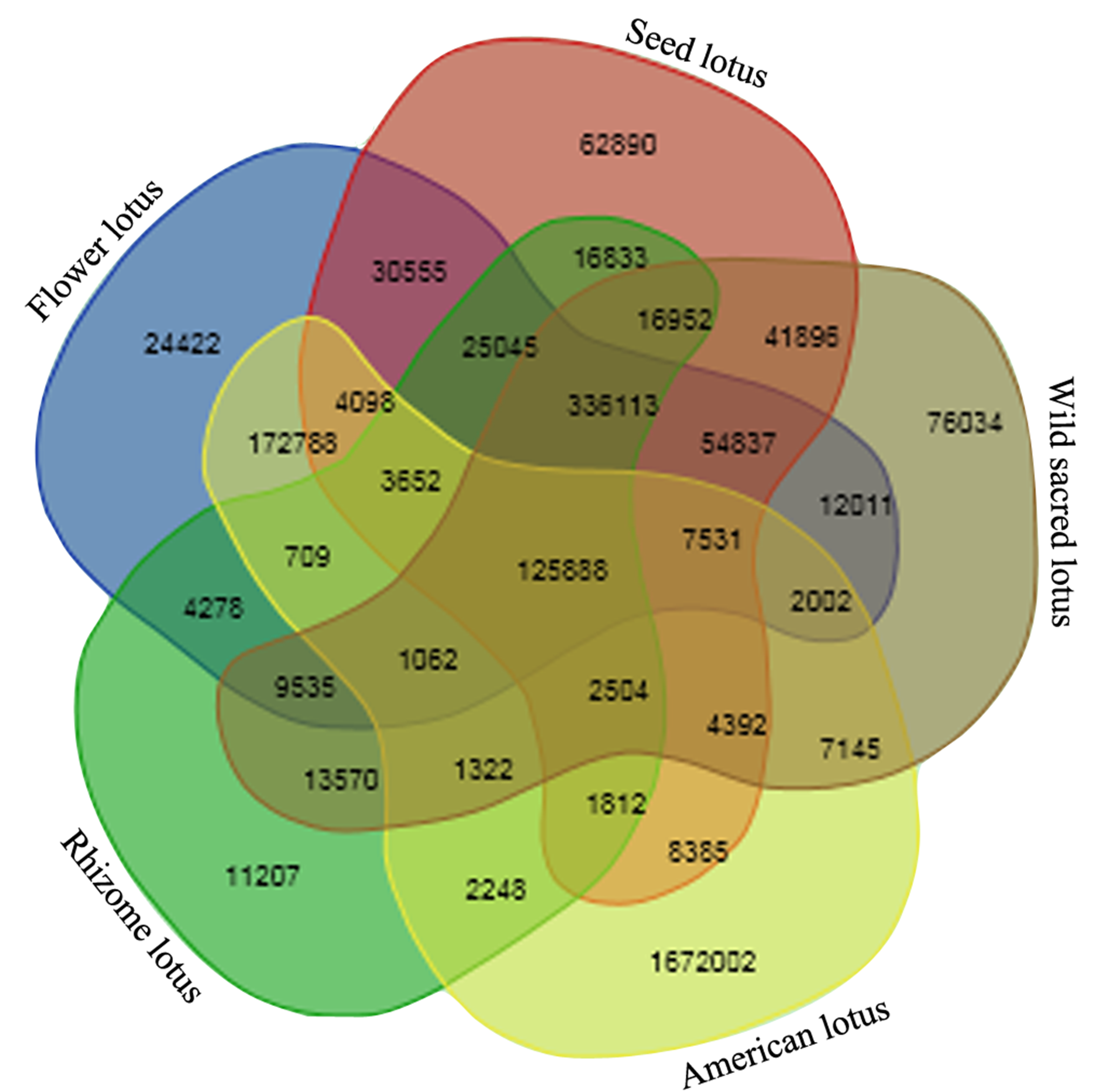

Supplement: Supplementary file 8 — Additional file 8: Figure. S3. Venn diagrams of the unique and common short insertion and deletions (indels) in the five groups. The individual and overlapping areas in the Venn diagrams represent the number of unique or common indels among the lotus groups. [file 12864_2019_6376_MOESM8_ESM.tif]

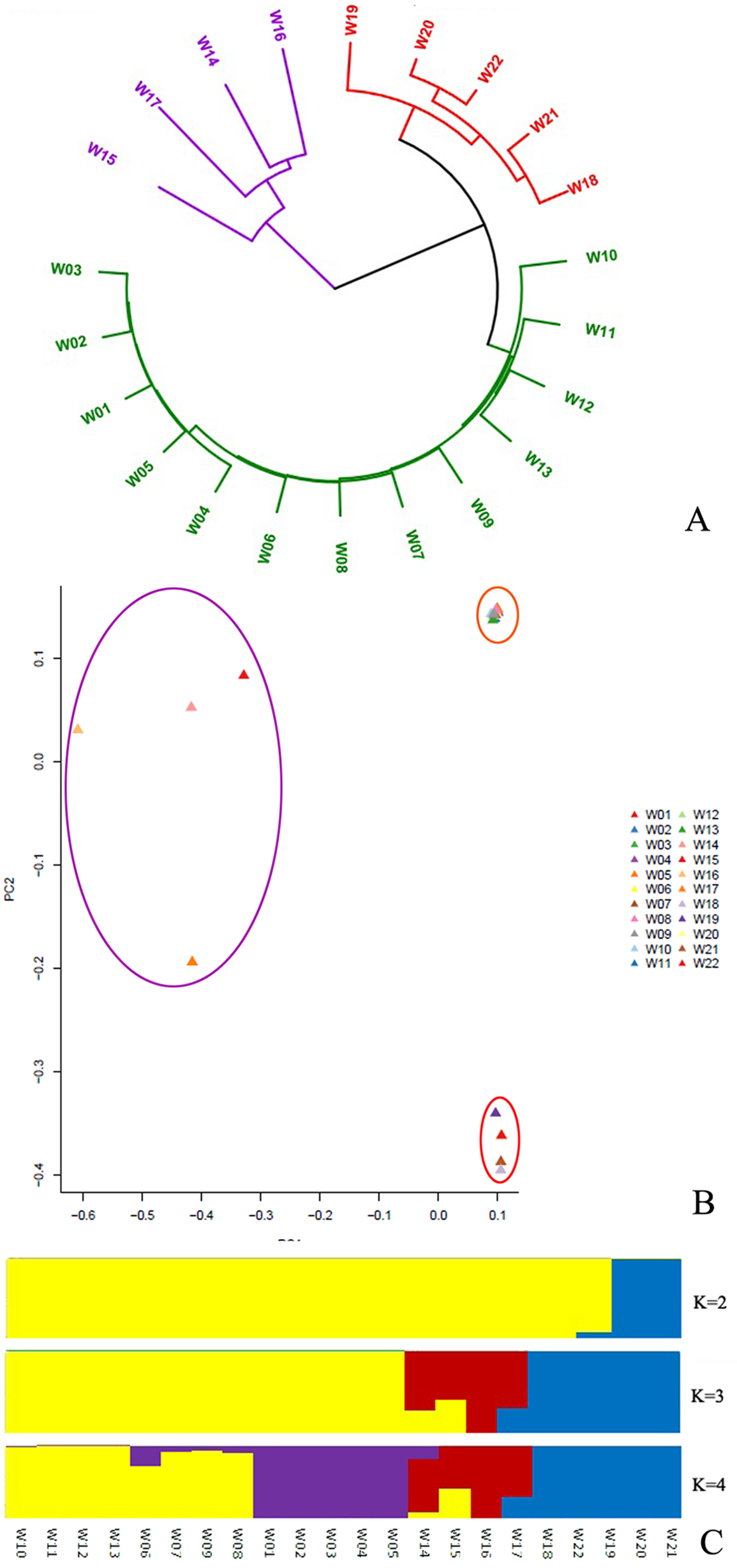

Supplement: Supplementary file 9 — Additional file 9: Figure S4. Analysis of the phylogenetic relationships and population structure of wild sacred lotus. (A) Neighbor-joining phylogenetic tree constructed using SNP data. (B) Principal component analysis (PCA) of wild sacred lotus. (C) Bayesian clustering (STRUCTURE, K = 2-4) of wild sacred lotus. [file 12864_2019_6376_MOESM9_ESM.tif]

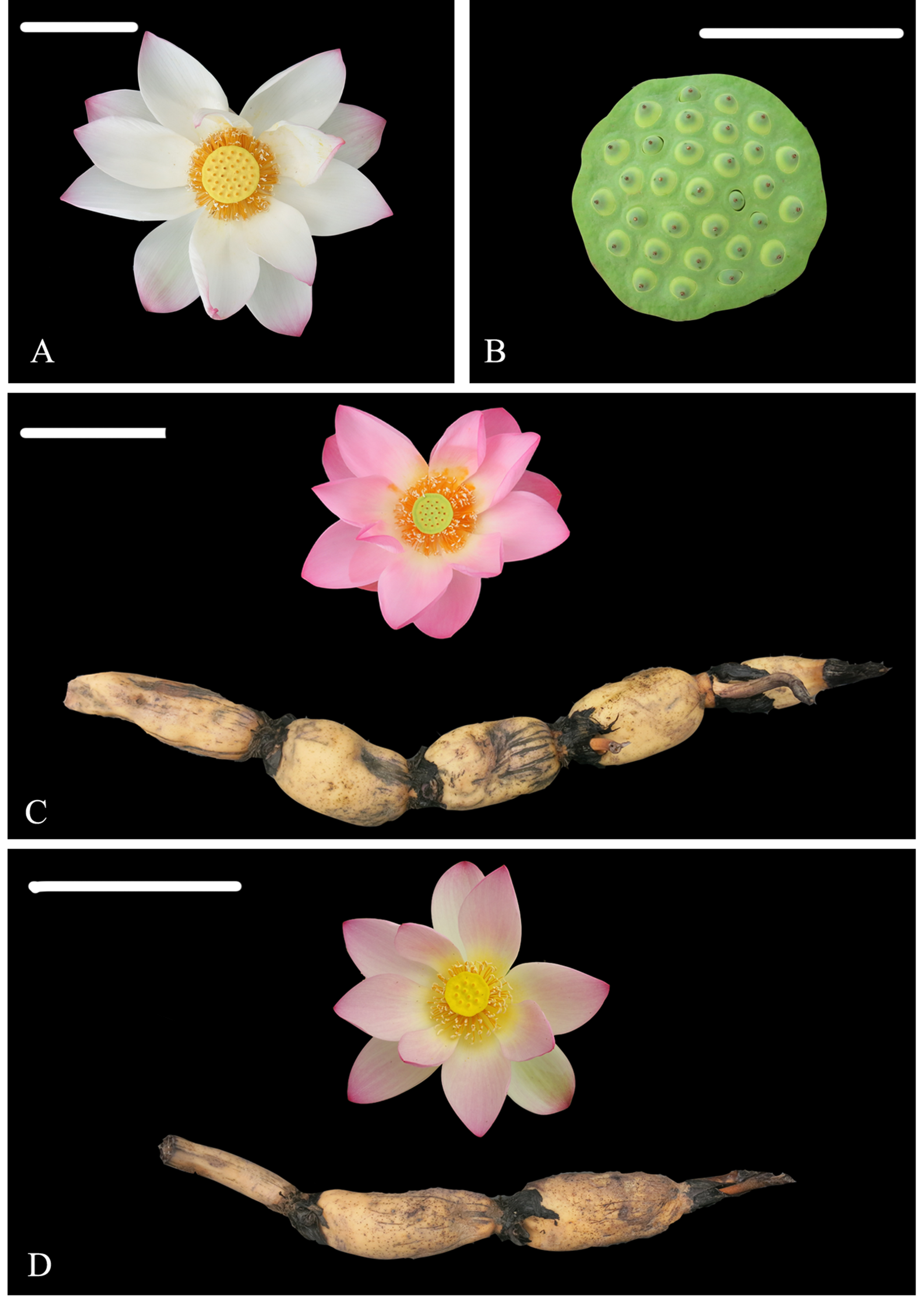

Supplement: Supplementary file 10 — Additional file 10: Figure S5. Morphology of the three lotus accessions based on multiple traits. Flower lotus accession F04 (A and B) exhibits both attractive flowers and many seeds per seedpod. Rhizome lotus accession R05 (C) exhibits both large rhizomes and many carpels per receptacle. Flower lotus accession F05 (D) exhibits both attractive flowers and swollen rhizomes. Bar indicates 10 cm. [file 12864_2019_6376_MOESM10_ESM.tif]

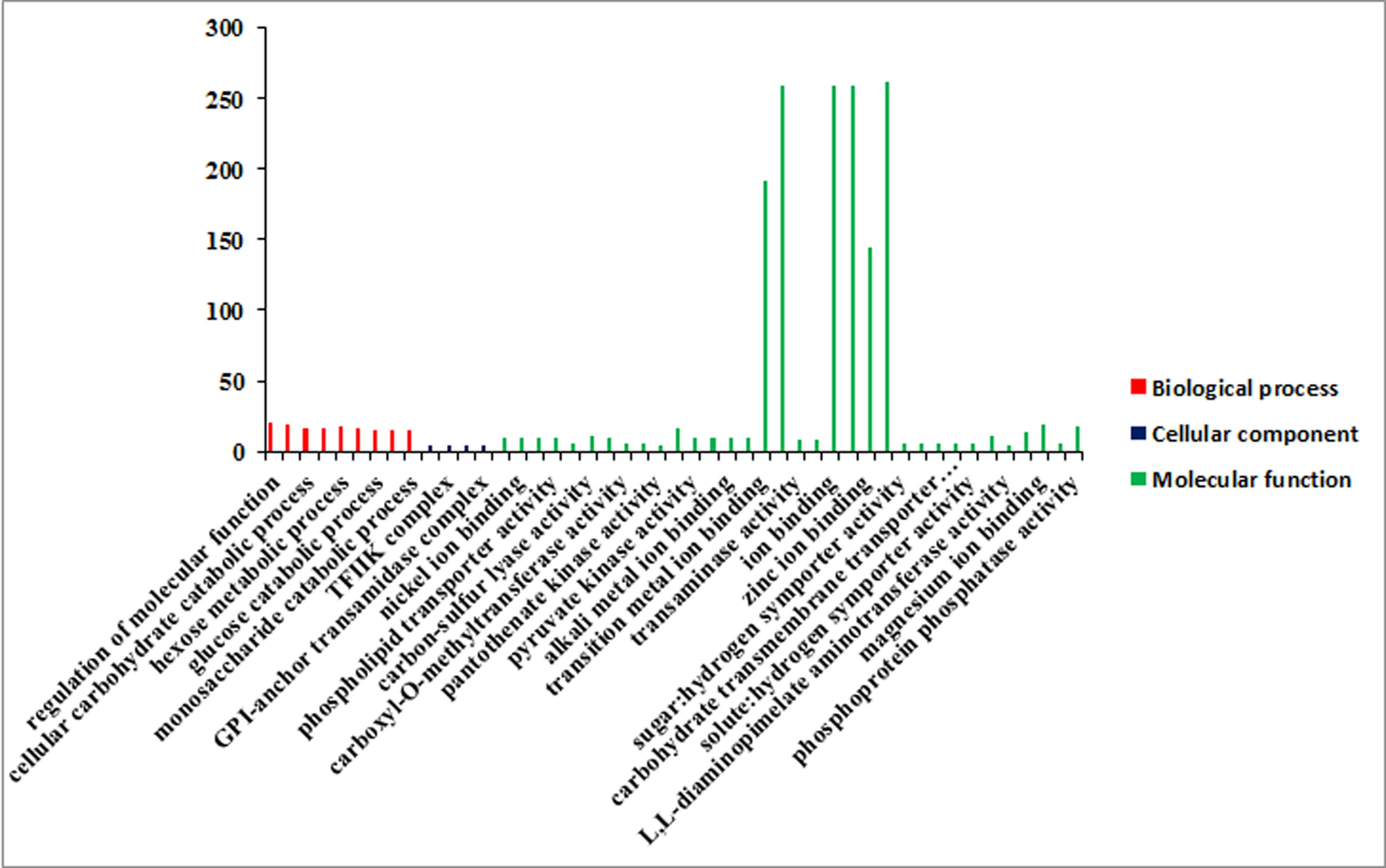

Supplement: Supplementary file 12 — Additional file 12: Figure S6. Gene Ontology analysis of the genes under artificial selection in seed lotus. [file 12864_2019_6376_MOESM12_ESM.tif]

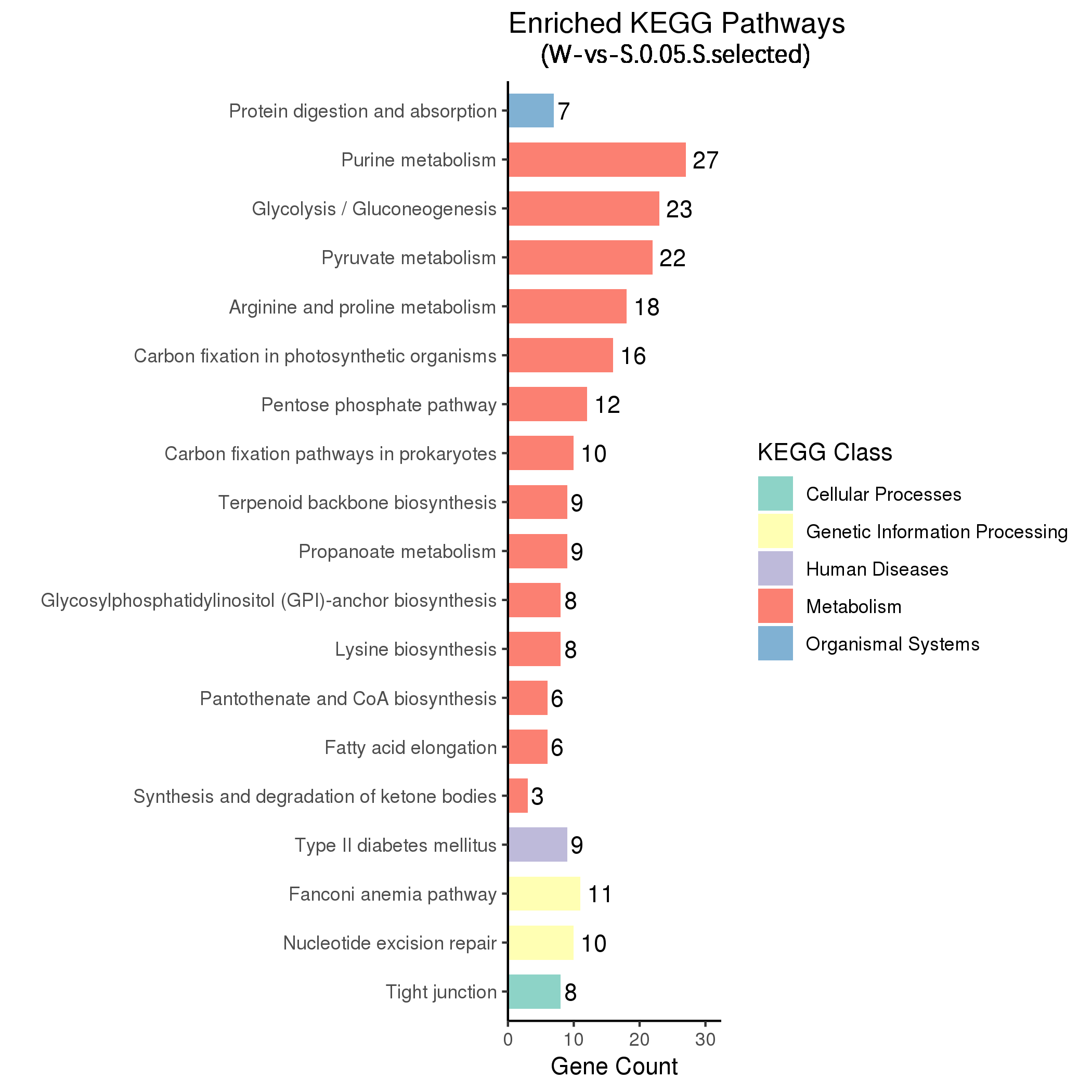

Supplement: Supplementary file 13 — Additional file 13: Figure S7. Kyoto Encyclopedia of Genes and Genomes analysis of the genes under artificial selection in seed lotus. [file 12864_2019_6376_MOESM13_ESM.tif]

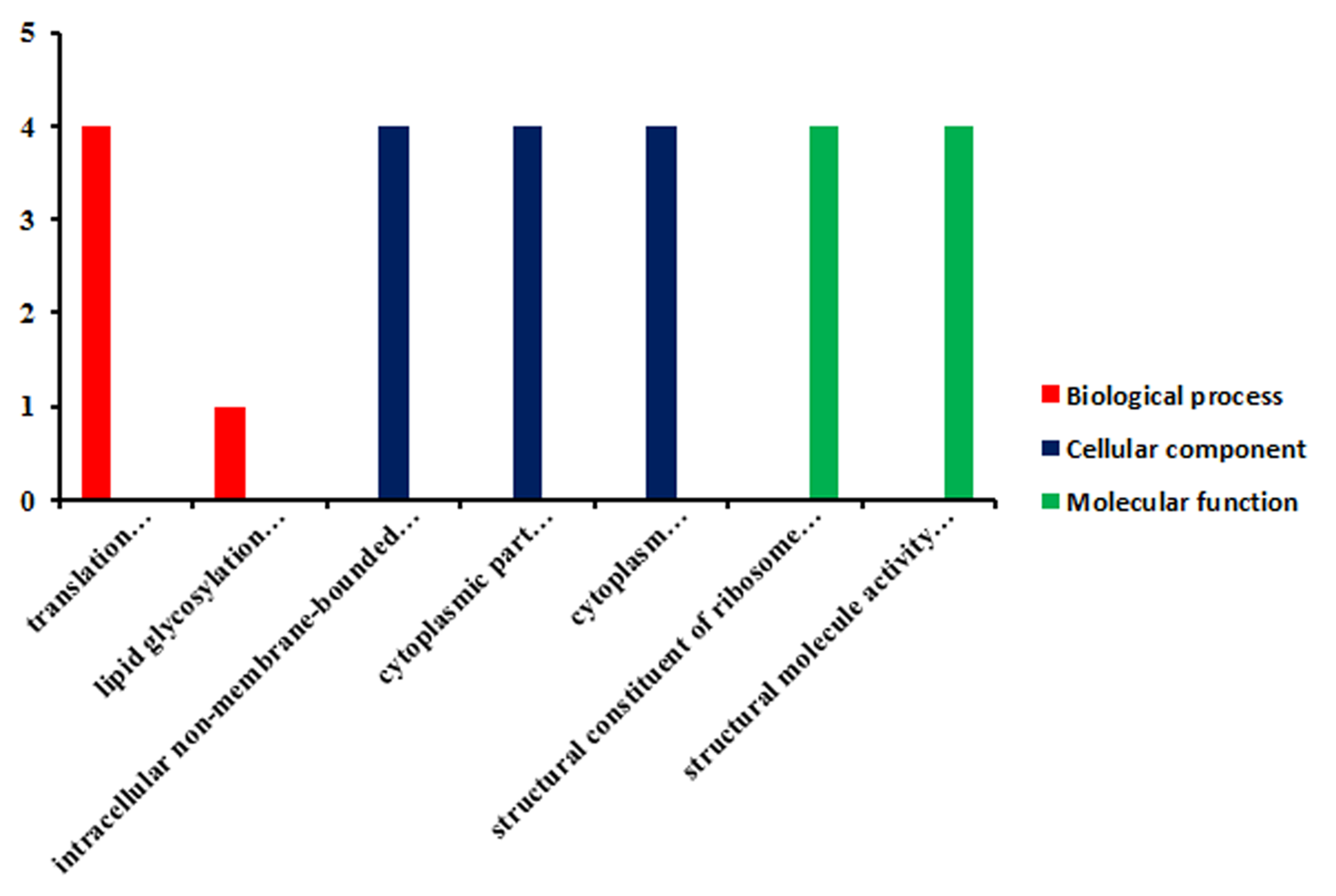

Supplement: Supplementary file 14 — Additional file 14: Figure S8. Gene Ontology analysis of the genes under artificial selection in flower lotus. [file 12864_2019_6376_MOESM14_ESM.tif]

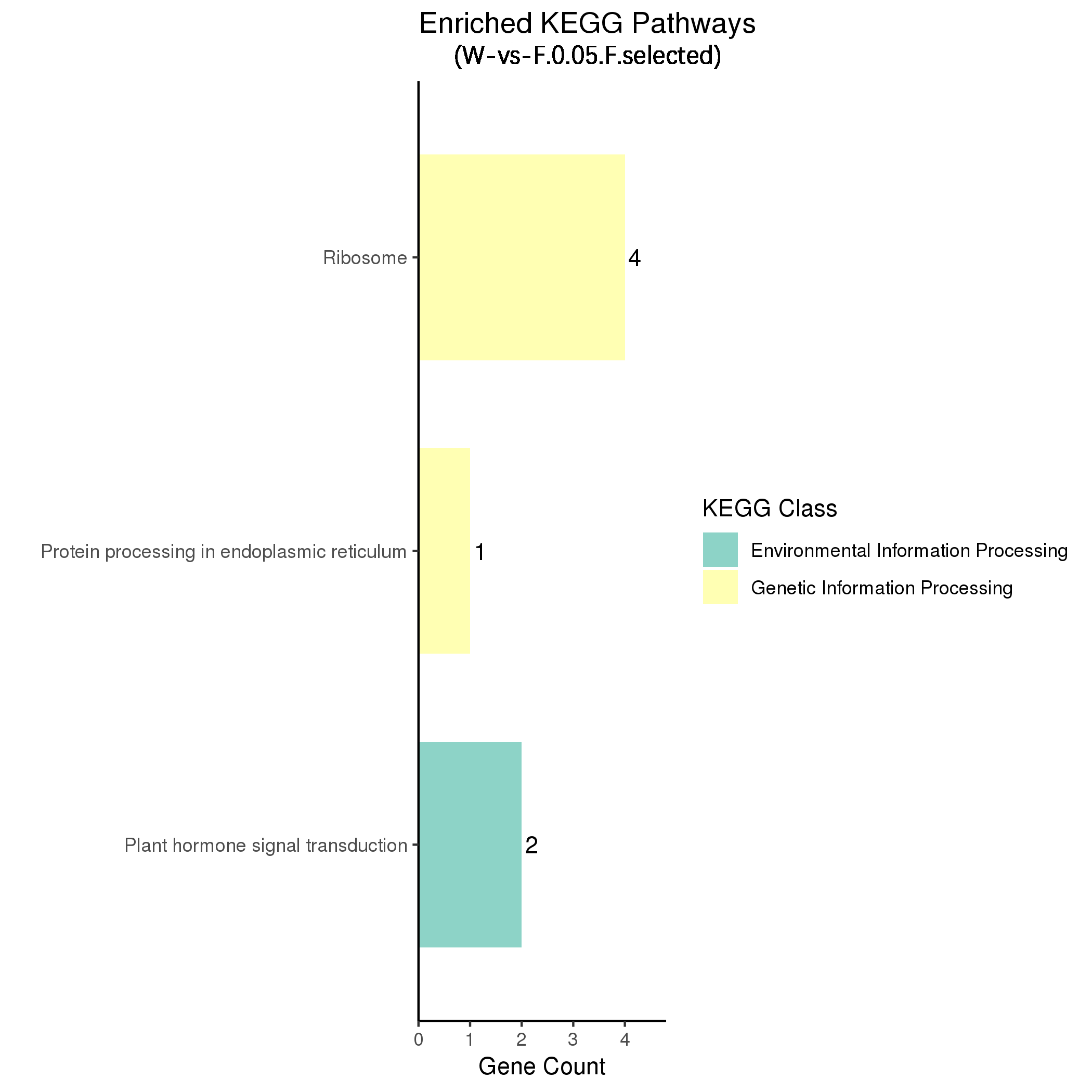

Supplement: Supplementary file 15 — Additional file 15: Figure S9. Kyoto Encyclopedia of Genes and Genomes analysis of the genes under artificial selection in flower lotus. [file 12864_2019_6376_MOESM15_ESM.tif]

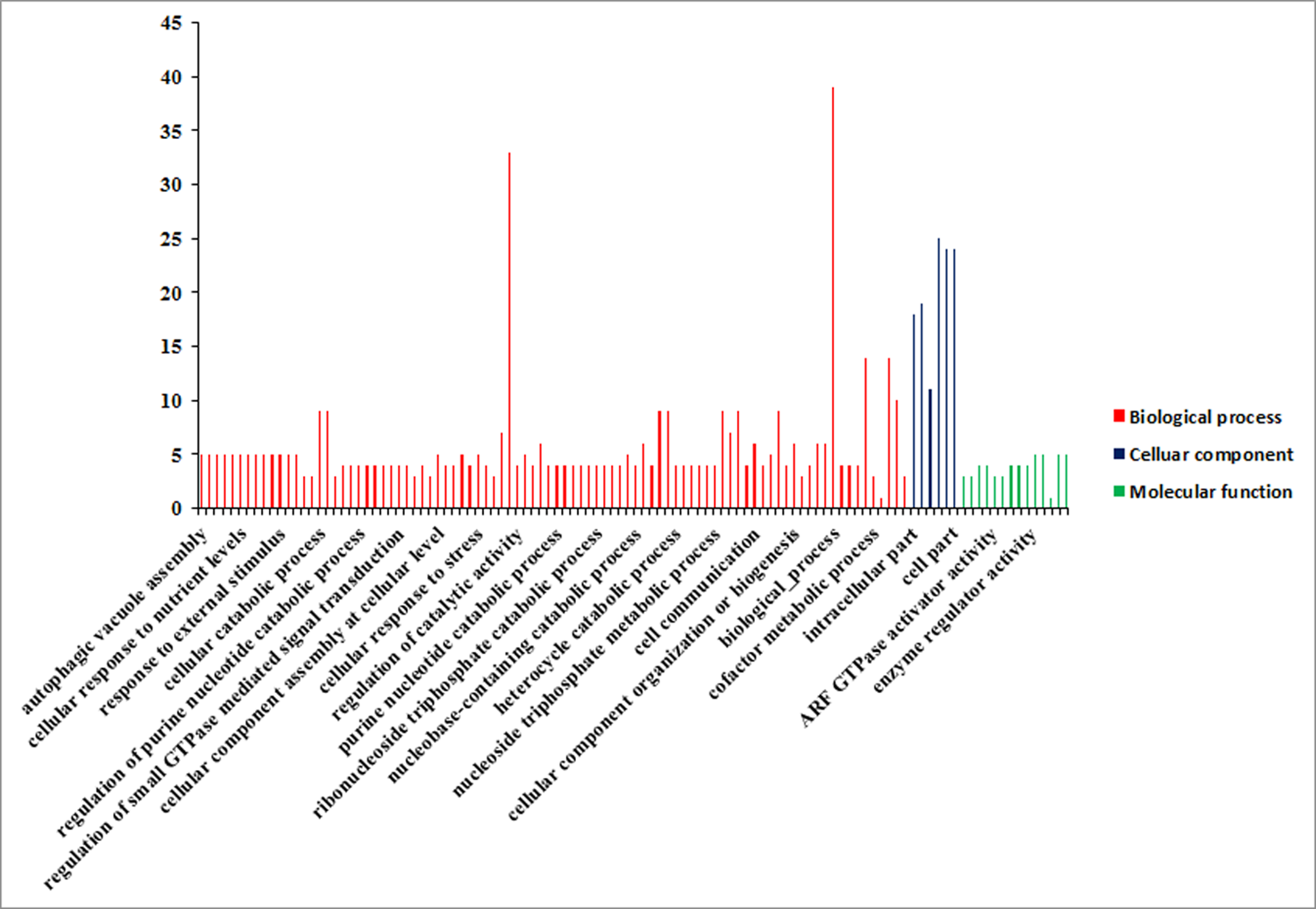

Supplement: Supplementary file 16 — Additional file 16: Figure S10. Gene Ontology analysis of the genes under artificial selection in rhizome lotus. [file 12864_2019_6376_MOESM16_ESM.tif]

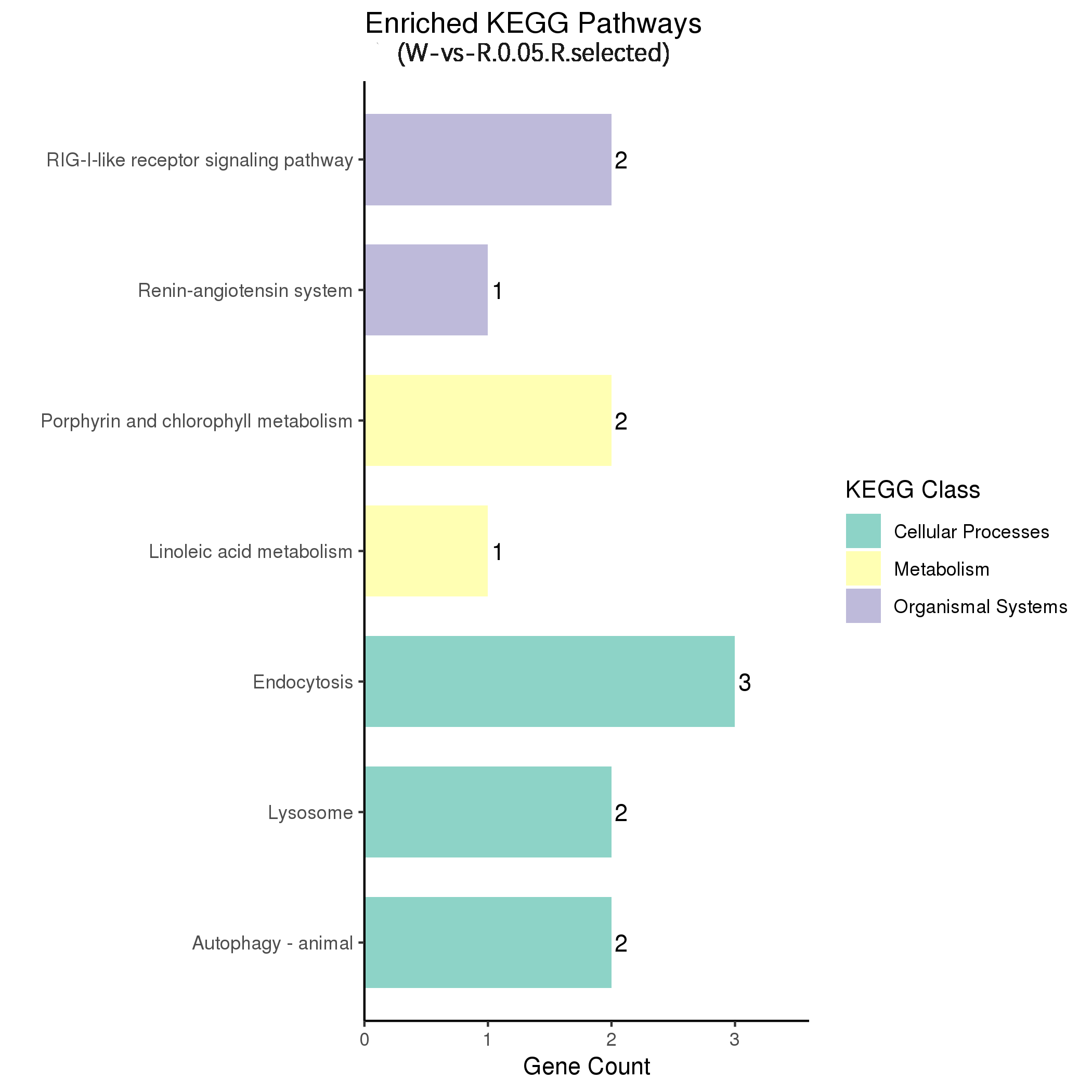

Supplement: Supplementary file 17 — Additional file 17: Figure S11. Kyoto Encyclopedia of Genes and Genomes analysis of the genes under artificial selection in rhizome lotus. [file 12864_2019_6376_MOESM17_ESM.tif]
